# Novel PCR-Based Detection Methods for the Lettuce Bacterial Leaf Spot Pathogen, *Xanthomonas hortorum* pv. *vitians* Morinière et al., 2020

**DOI:** 10.3390/plants14060964

**Published:** 2025-03-19

**Authors:** Emma R. Martinez, Mozhde Hamidizade, Ana B. Zacaroni, Carolee T. Bull

**Affiliations:** 1 Department of Plant Pathology and Environmental Microbiology, Pennsylvania State University, University Park, PA 16802, USA; emma.martinez@usda.gov (E.R.M.); mxh6128@psu.edu (M.H.); 2 Embrapa Genetic Resources and Biotechnology, Federal District, Brasilia 70770-917, Brazil; anabeatriz.zacaroni@gmail.com; 3 Department of Plant and Soil Sciences, Faculty of Natural and Agricultural Sciences, University of Pretoria, Pretoria 0083, South Africa

**Keywords:** disease detection, bacterial disease, genetic diversity, comparative genomics, PCR-based detection, *Lactuca sativa*

## Abstract

Bacterial leaf spot in lettuce is a sporadic but devastating disease that threatens lettuce production worldwide. Severe outbreaks have resulted in up to 100% crop loss, and even smaller outbreaks can cause a significant yield loss, as the affected tissue must be removed from lettuce heads prior to their sale. The pathogen, *Xanthomonas hortorum* pv. *vitians* (*Xhv*), has at least three races, with each defined by the disease or resistance phenotype it elicits in lettuce cultivars and accessions. Specific molecular detection of *Xhv* would facilitate the work of clinicians, growers, seed companies, and researchers in the lettuce industry. We present an *Xhv*-specific touchdown PCR method and progress toward race-specific methods. We used an alignment of 18 *Xhv* strains and 9 closely related, non-target strains to identify pathovar- and race-specific gene clusters as targets for PCR primers. We evaluated the specificity first using in silico methods and then empirically using a collection of *Xanthomonas* strains. Our protocol demonstrated *Xhv*-specific detection from two sample types, including genomic DNA extracts and bacterial suspensions. Additional research is required to refine the race-specific protocols.

## 1. Introduction

Bacterial leaf spot in lettuce poses a significant threat to the 4.1-billion-dollar lettuce industry in the United States [1]. Most of its large-scale production occurs in California, Arizona, and Florida, and this disease has been detected in these areas and worldwide where lettuce is grown [2,3]. Nearly all market lettuce is cultivated for its leaves, which are damaged in a bacterial leaf spot infection due to the disease’s symptoms: water-soaking, chlorosis, and small necrotic spots that later join to form large lesions [4]. Following an outbreak, crop loss and the removal of infected leaves from the surviving lettuce heads result in a sizable reduction in both the sellable yield and product value. Cool, wet weather favors disease development, and although sporadic, outbreaks have been shown to affect up to 100% of field-grown lettuce [5].

The successful management of any plant disease depends on the timely and accurate identification and detection of the pathogen. Bacterial leaf spot in lettuce is caused by a bacterium, *Xanthomonas hortorum* pv. *vitians* (*Xhv*, formerly *X. campestris* pv. *vitians* or *Xcv*) [6]. Previously, a conventional PCR was developed by Barak et al. [7]. This method using B162 primers resulted in target amplification from *Xcv* strains but not from the other *X. campestris* strains tested, suggesting that the protocol was *Xcv*-specific. However, *Xcv* and other members of *X. campestris* have since been transferred to *X. hortorum* as *Xhv*, *X. hortorum* pv. *carotae*, *X. hortorum* pv. *cynarae*, *X. hortorum* pv. *gardneri*, *X. hortorum* pv. *hederae*, *X. hortorum* pv. *pelargonii*, and *X. hortorum* pv. *taraxaci* [6,8]. Of the closely related pathovars, Barak et al. [7] only included *X. hortorum* pv. *carotae* and *X. hortorum* pv. *pelargonii* as outgroup strains during the development of their method. In this manuscript, we demonstrate that in addition to *Xhv*, the B162-based conventional PCR amplifies several other *X. hortorum* pathovars and closely related strains, though not *X. hortorum* pv. *carotae* and *X. hortorum* pv. *pelargoni*. This lack of specificity could lead to false positive results when seeking to identify or detect *Xhv*. We sought to develop a conventional PCR that would replace this method for the identification and detection of *Xhv*.

Similarly, *Xhv* can be detected using the loop-mediated isothermal amplification (LAMP) assay developed by Dia et al. [9], but its detection is not limited to *Xhv* strains. Instead, this method targets the clade containing *Xhv*, *X. hortorum* pv. *gardneri*, and *X. hortorum* pv. *cynarae* [9]. The continued lack of an *Xhv*-specific method among the previously published detection methods prompted our research to fill this technical gap.

The phenotypic variation among *Xhv* strains is an important consideration in the development of detection methods. *Xhv* consists of three distinct groups of strains capable of triggering a host hypersensitive response (HR), each in a different lettuce cultivar or accession [10,11]. The HR is a plant defense strategy; after the recognition of a pathogen, programmed cell death of the affected tissue is initiated to limit the spread of infection. It has been hypothesized that this variation in plant reactions to *Xhv* strains, used to define the three groups as “races”, is caused by genetic variation. This claim has been supported by both a multilocus sequence analysis [10] and a genome-wide SNP variant analysis [12]; in both cases, the *Xhv* strains were clustered by race. A set of race-specific PCR-based detection methods would need to target sequences common to strains within a single known race and absent from non-target, related strains.

Following this strategy, we developed touchdown PCR protocols based on the whole genome alignments of 18 *Xhv* strains and 9 closely related strains. The primers were designed to target gene clusters present in all of the *Xhv* strains but absent from all of the others. We originally designed a conventional PCR, but we found in testing subsets of strains that the touchdown method improved the specificity of the reactions. We then tested our protocol using a collection of 96 *Xhv* isolates from around the world representing the three known races, the 9 other closely related strains within *X. hortorum* and *X. hydrangeae*, and 25 plant pathogenic type strains within *Xanthomonas*. Once we observed the desired *Xhv* specificity with this collection, we evaluated our method using two *Xhv* isolates from a 2018 bacterial leaf spot outbreak in lettuce in Pennsylvania, which were correctly detected using our touchdown method. The same process was used to develop methods for race-specific detection; we observed mixed results due to the absence of target amplification, or the presence of non-target amplification, but here, we share our progress and insights for future development.

## 2. Results

### 2.1. The Specificity of the Previously Published Detection Methods

**Figure 1:**
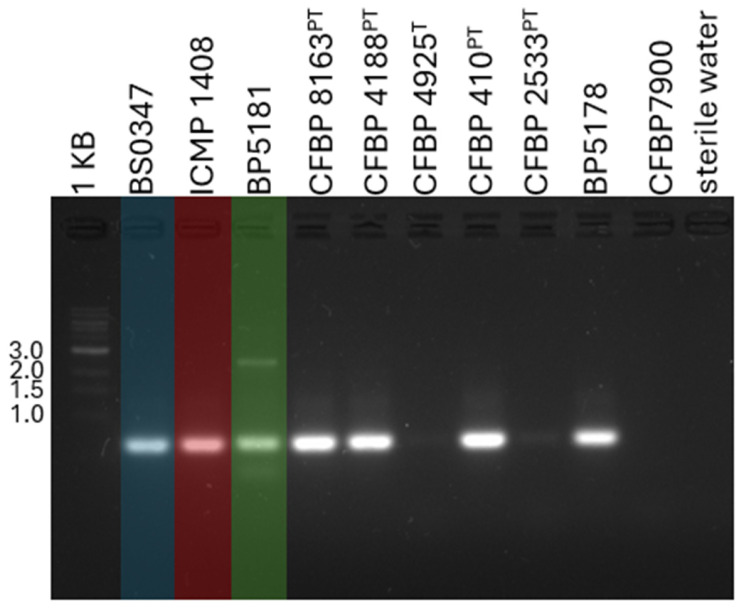
**Conventional PCR using B162 primers.** The agarose gel shows the banding pattern upon the amplification of a 700 bp fragment using B162 primers [7] and *X. hortorum* genomic DNA as the templates. *Xhv* strains are included with overlays in blue (BS0347; race 1), red (ICMP 1408; race 2), and green (BP5181; race 3); the other strains included are *Xanthomonas hortorum* pv. *gardneri* (CFBP 8163^PT^), *X. hortorum* pv. *cynarae* (CFBP 4188^PT^), *X. hortorum* pv. *hederae* (CFBP 4925^T^), *X. hortorum* pv. *taraxaci* (CFBP 410^PT^), *X. hortorum* pv. *pelargonii* (CFBP 2533^PT^), *X. hortorum* from radicchio (BP5178), and *X. hortorum* pv. *carotae* (CFBP 7900). The first well includes a 1 KB ladder, and the final well was run with sterile water as a template.

When we tested the B162 primer set developed by Barak et al. for *Xhv*-specific detection [7], we found that while it resulted in the amplification of the targeted 700-base-pair (bp) fragment from the *Xhv* strains (BS0347, race 1; ICMP 1408, race 2; BP5181, race 3), as expected, it also amplified the same fragment from four non-target yet closely related strains: *X. hortorum* pv. *garderi* (CFBP 8163^PT^), *X. hortorum* pv. *cynarae* (CFBP 4188^PT^), *X. hortorum* pv. *taraxaci* (CFBP 410^PT^), and an *X. hortorum* strain isolated from radicchio (BP5178) (Figure 1). The target fragment was not amplified from the other closely related strains, *X. hortorum* pv. *hederae* (CFBP 4925^T^), *X. hortorum* pv. *pelargonii* (CFBP 2533^PT^), and *X. hortorum* pv. *carotae* (CFBP 7900).

These results were corroborated by our in silico data. The B162 amplicon encoded a glycosyl hydrolase of family 3 that may play a role in starch hydrolysis, toxin degradation, and virulence promotion [13], and when we compared it to the NCBI whole genome database, it shared high sequence homology with a region in the whole genomes of the *X. hortorum* pvs. *vitians*, *gardneri*, *cynarae*, and *taraxaci* strains, as well as with a region in another close relative, *X. campestris* pv. *nigromaculans*, a pathogen of the Eurasian root vegetable and otherwise invasive weed known as greater burdock [14] (Supplemental Figure S1). Such high sequence homology could explain the ability of the B162 primers to bind to the off-target *X. hortorum* strains’ genomic DNA; further, these results suggested that *X. campestris* pv. *nigromaculans* could also be detected using this method. There were other *X. campestris*, *X. arboricola*, and *Xyllela fastidiosa* strains among the matches with the 700 bp B162 amplicon sequence, but they had significant variation that would likely prevent primer binding and subsequent target amplification from these strains.

Our laboratory and in silico experiments demonstrated that the conventional PCR procedure using the B162 primers does not have sufficient specificity to distinguish *Xhv* from several other agriculturally significant *Xanthomonas* plant pathogens, including many closely related *X. hortorum* pathovars. These results revealed the need for an improved, pathovar-specific protocol for *Xhv* detection that would remove the risk of false positive results in agricultural settings where multiple plant pathogens may be present.

### 2.2. A Pangenome Analysis Revealed Xhv Pathovar- and Race-Specific Gene Clusters

Pangenome alignment revealed gene clusters that were unique to the *Xhv* strains and not present in any of the genomes from the other *Xanthomonas* strains included in the alignment. Several other gene clusters appeared to be race-specific (encoded by all strains of a single *Xhv* race but not by the *Xhv* strains of the other races). A full list of the identified gene clusters is given in Supplemental Table S1, but our in silico evaluations of specificity using NCBI’s BLASTs led us to select gene clusters 3906, 4021, 4381, and 4980 to evaluate their specificity for *Xhv*, *Xhv* race 1, *Xhv* race 2, and *Xhv* race 3, respectively.

We chose gene cluster 3906 (GC3906) for *Xhv* specificity. For this gene cluster, we found no matches with NCBI’s database of conserved protein domains collected from most known forms of life. Comparing the gene cluster sequence to the NCBI BLAST database revealed that it had a 100% identity match with a hypothetical protein in *Xhv* but only a 90–98% identity match with the same protein in *X. arboricola*, *X. nasturtii*, *X. campestris*, *X. phaseoli*, and *X. translucens* (Supplemental Figure S2). Due to the observed sequence variation in all of the non-*Xhv* matches, it appeared to be a good candidate for the specific detection of *Xhv* strains. Comparisons of this cluster with the NCBI whole genome database revealed hits for the same *Xhv* strains that we included in our pangenome alignment, as expected, but also for several other *X. hortorum* strains that lack pathovar designations. *X. hortorum* pv. *gardneri* Xc69, which was re-classified from *X. arboricola* based on the average nucleotide identity (ANI) and genome-based DNA-DNA (gDDH) hybridization data [15], also appeared as a match. However, the ANI and gDDH are typically used for species determination and are not precise enough to distinguish between pathovars within the same species. While this strain may be a member of *X. hortorum*, its pathovar designation remains unclear. Two *Xhv* strains of unknown races and all of the known *Xhv* race 2 strains for which we had previously sequenced whole genomes [12] included the same SNP in this gene cluster, while all of the other matches described above were identical. All additional matches were not from *Xhv* strains and had significant sequence variation, including strains of *X. nasturtii*, *X. campestris*, and *X. hortorum* pv. *cynarae*. These results indicated that an *Xhv*-specific PCR-based detection method could be designed using the GC3906 gene cluster if the primers were designed to bind to segments that were conserved in all *Xhv* strains but showed variation in non-target strains.

Gene clusters that were unique among the *Xhv* strains to one of the three races were not necessarily absent from the related *X. hortorum* pathotype strains or other reference strains. Any race-specific detection method would need to be used in tandem with a pathovar-specific protocol for *Xhv* detection. For race 1, we investigated gene cluster 4021 (GC4021). We found a 100% identity match with an *Xhv* helix–turn–helix transcriptional regulator domain which is known to be activated in bacteria as a response to chemical stressors [16] (Supplemental Figure S3). This cluster matched with all *Xhv* race 1 strains for which we had previously obtained whole genome sequences [12] and no other *Xhv* strains. Many *X. hortorum* pv. *pelargonii* strains and one *X. gardneri* strain also matched but with sequence variations and truncations that could be exploited during the primer design to develop a *Xhv* race 1-specific reaction.

Neither GC4381 nor GC4980 had any matches with conserved domains, and each had a 100% identity match with different hypothetical proteins from *Xhv* (Supplemental Figures S4 and S5). GC4381 had identical sequence matches to all four *Xhv* race 2 strains that we had sequenced previously [12] and two *Xhv* strains of unknown races that we predict to also be *Xhv* race 2 strains. GC4980 had identical sequence matches to the two *X. hortorum* pv. *vitians* race 3 strains that were sequenced previously [12]. We therefore utilized GC4381 and GC4980 in an attempt to develop race 2- and race 3-specific primers, respectively.

### 2.3. The Development of an Xhv-Specific Detection Method

**Figure 2:**
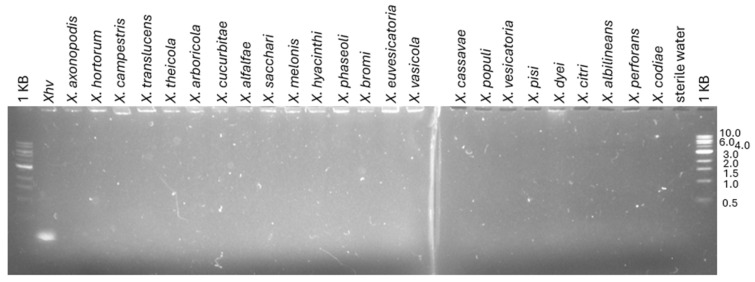
**Evaluation of our *Xhv*-specific method using twenty-four *Xanthomonas* type strains.** Genomic DNA from the following strains was used as templates for touchdown PCR using our GC3906-152 primer set designed for *Xhv*-specific detection: *Xhv* (CFBP 8686^PT^), *X. axonopodis* (CFBP 4924^T^), *X. campestris* (CFBP 5251^T^), *X. translucens* (CFBP 2054^T^), *X. theicola* (CFBP 4691^T^), *X. arboricola* (CFBP 2528^T^), *X. cucurbitae* (CFBP 2542^T^), *X. alfalfae* (CFBP 7686^T^), *X. sacchari* (CFBP 4641^T^), *X. melonis* (CFBP 4644^T^), *X. hyacinthi* (CFBP 1156^T^), *X. phaseoli* (CFBP 8462^T^), *X. bromi* (CFBP 1976^T^), *X. euvesicatoria* (CFBP 6864^T^), *X. vasicola* (CFBP 2543^T^), *X. cassavae* (CFBP 4642^T^), *X. populi* (CFBP 1817^T^), *X. vesicatoria* (CFBP 2537^T^), *X. pisi* (CFBP 4643^T^), *X. dyei* (CFBP 7245^T^), *X. citri* (CFBP 3369^T^), *X. albilineans* (CFBP 2523^T^), *X. perforans* (CFBP 7293^T^), and *X. codiae* (CFBP 4690^T^). The 1 KB DNA ladder from NEB was used as a size reference for the amplicon, and 1% agarose gels were run for 20 min at 120 V.

Many primer sets were designed to target the *Xhv*-specific gene clusters identified through the pangenome alignment, and while all of the others were eliminated during empirical testing due to the detection of off-targets, GC3906-152 (so called after the targeted gene cluster and amplicon size) proved to be an effective target for *Xhv*-specific detection (Table 1). The target fragment was amplified from 99% (95 out of 96) of the *Xhv* strains tested, including the pathotype strain CFBP 8686^PT^ (Table 2; Supplemental Figure S6). However, the target fragment was not amplified from strain BS3126, a race 2 strain from France. The target fragment was not detected from any of the other *X. hortorum* pathotype strains, *X. hydrangeae* (LMG 31884^T^), *X. campestris* pv. *coriandri* (CFBP 8452^PT^), or *X. hortorum* from radicchio (BP5178) [17], or any of the 25 *Xanthomonas* species type strains included in this study, resulting in 100% exclusivity (Figure 2; Table 2). No amplification was observed for any of the sterile water controls.

### 2.4. Evaluating the Xhv-Specific Detection Method Using Environmental Samples

**Figure 3:**
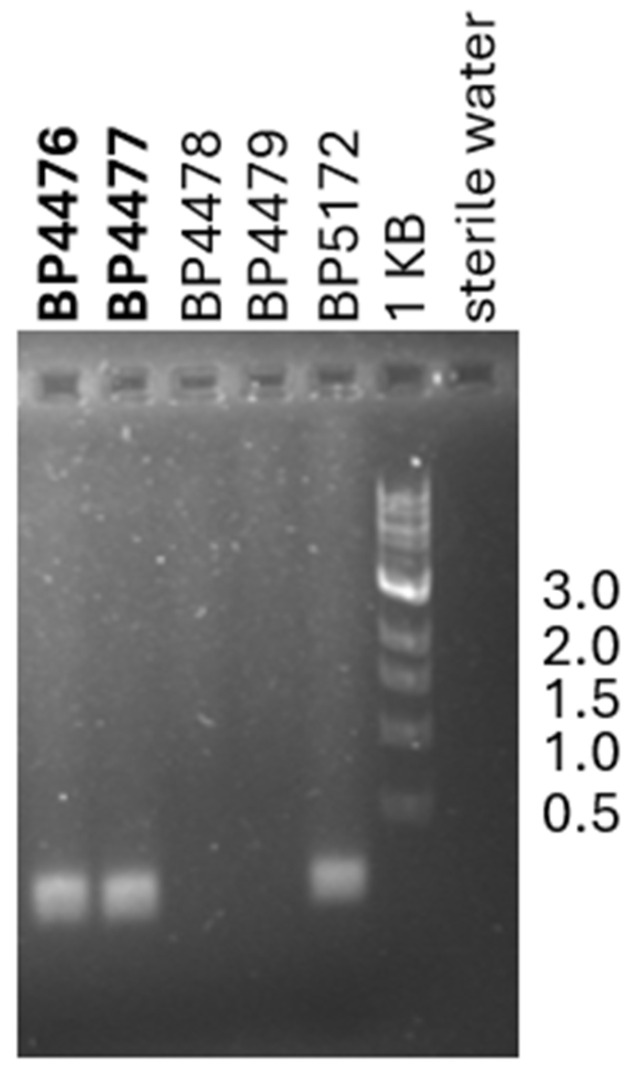
Evaluation of our *Xhv*-specific detection method using environmental samples collected from a 2018 bacterial leaf spot outbreak in lettuce in Pennsylvania. Two *Xhv* isolates, BP4476 and BP4477, and two *Pseudomonas* isolates, BP4478 (*Pseudomonas viridiflava*) and BP4479 (*Pseudomonas allivorans*), isolated from the same symptomatic lettuce tissue were used as the templates in the *Xhv*-specific touchdown reaction with the GC3906-152 primers to test the primers’ specificity. *Xhv* race 1 strain BP5172 was included as a positive control, sterile water was included as an experimental control, and the size reference was a 1 KB DNA ladder from NEB. The 1% agarose gel was run for 20 min at 120 V.

**Figure 4:**
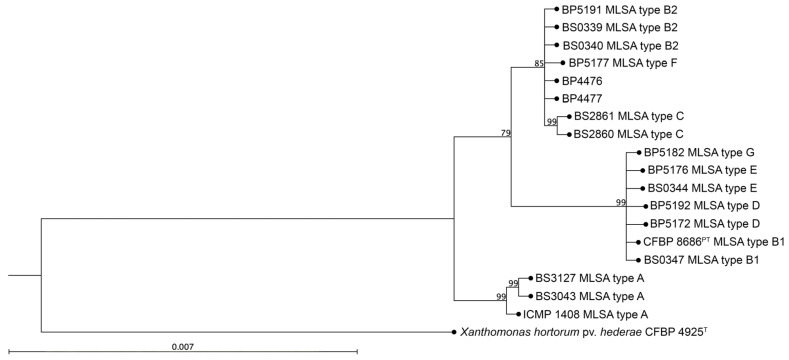
**Multilocus sequence analysis (MLSA) of PA *X. hortorum* isolates.** Maximum likelihood phylogeny generated from the alignment of concatenated fragments of the *rpoD*, *fyuA*, *gyrB*, and *gap1* housekeeping genes using the neighbor-joining algorithm and the general time-reversible nucleotide substitution model (including rate variation and estimated topology). The bootstrap threshold was set to 70%, and the branch lengths represent the expected number of nucleotide substitutions per site. Branches shorter than 0.0002 are shown as having a length of 0.0002. Strains of a known *Xhv* MLSA type were included as a reference set in order to predict the races of the Pennsylvania strains that tested positive for *Xhv* detection using our touchdown PCR-based method. The type strain of *X. hortorum* pv. *hederae* was included as an outgroup.

To evaluate whether the *Xhv*-specific PCR protocol designed could be used to identify *Xhv* from novel outbreaks, four isolates collected from a 2018 outbreak of BLS in lettuce in Pennsylvania were investigated. The *Xhv*-specific touchdown protocol using the GC3906-152 primer set amplified fragments from only two of the four strains. The expected 152 bp band was amplified for BP4476 and BP4477 but not for BP4478 and BP4479 (Figure 3). As expected, the positive control strain, *Xhv* race 1 BP5172, produced the expected 152 bp band, and the sterile water control did not. Strains BP4476 and BP4477 were identified as *Xhv* using the MLSA, which placed them within the clade containing the *Xhv* race 1 strains (Figure 4). Analysis of the 16S rRNA subunit sequence revealed that the two other isolates, BP4478 and BP4479, were members of *Pseudomonas viridiflava* (99.9% identity and 100% query cover) and *Pseudomonas allivorans* (100% identity and 100% query cover), respectively.

### 2.5. Progress Toward Xhv Race-Specific Detection

Many gene clusters were also evaluated as candidate targets for *Xhv* race-specific detection using our touchdown PCR method. The best-performing primer sets, those with the greatest number of target strains detected and the least number of non-targets, were selected and are shown in Table 1. These included GC4021-112, GC4381-138, and GC4980-178 for *Xhv* race 1-, 2-, and 3-specific detection, respectively. When used with our touchdown method, GC4021-112 resulted in strong amplification of a 112 bp fragment from 48 strains (59%) and its faint amplification from 2 strains (2%) out of the 82 strains that either demonstrated HR elicitation in the *L. sativa* cultivar Little Gem, leading to an *Xhv* race 1 designation, or clustered with the known *Xhv* race 1 strains in the MLSA, leading to the prediction that they belonged to *Xhv* race 1 (Table 2; Supplemental Figure S7). Among the thirty-four strains that were not detected using this method, two strains were known members of race 1 and were included in the pangenome alignments used to identify the race-specific gene clusters. This indicates that the whole genome sequence assemblies for these strains, BP5172 and BP5177, may not be representative of the true gene cluster sequence, possibly due to the limited ability of short reads to resolve certain types of genetic variations, such as long repetitive sequences. Such a consequence of the short-read assemblies may also have been true for the other twenty-eight known or predicted *Xhv* race 1 strains that did not show amplification of the 112 bp fragment. Long-read sequencing will be needed to evaluate these hypotheses and improve the positioning of the primers to improve the specificity or to identify stronger targets for race-specific detection. However, the protocol did successfully exclude all *Xhv* race 2 and 3 strains tested; none of these strains produced the 112 bp fragment, and this was expected, as the gene cluster GC4021 was absent from these strains’ alignments.

The same touchdown PCR protocol was used to evaluate the GC4381-178 primer set for *Xhv* race 2-specific detection. Among the race 2 strains tested, 8 out of 10 (80%) produced the expected amplicon, excluding BS3531 and BS3532 (Table 2; Supplemental Figure S8). The two strains that did not produce the amplicon were not included in the pangenome alignment used to identify the race-specific gene clusters because they lacked available whole genome sequence data. It is possible that these two strains, known to belong to race 2 due to their elicitation of an HR in *L. serriola* PI491114, do not encode GC4381 or that they encode a sufficient variation in this cluster to prevent primer binding. Other unexpected results included the detection of two known *Xhv* race 1 strains, one that was part of the pangenome alignment that showed GC4381 to be absent (BP5182) and one predicted to belong to *Xhv* race 1 based on the MLSA data (BS2996). It is possible that these strains retain GC4381 but that it is somehow inactive and unable to elicit an HR in *L. serriola* PI491114. These hypotheses will be investigated as we continue to improve the protocol; long-read sequencing will be needed to help resolve the conflicting results between the in silico sequence analyses and the empirical HR testing.

The touchdown PCR protocol used with the GC4980-138 primer set for *Xhv* race 3 detection showed amplification of the 138 bp target fragment in one known *Xhv* race 3 strain and three *Xhv* race 3 strains whose identities were predicted based on the MLSA data (Table 2; Supplemental Figure S9) [10]; these are all of the *Xhv* race 3 strains currently in curation. Non-target amplification of the 138 bp fragment occurred for *X. hortorum* pv. *carotae* CFBP 7900, though it was faint, which was part of our pangenome alignment, and for an *Xhv* strain predicted to belong to race 1 due to the MLSA results (BS3272). In combination with this protocol, the use of the described pathovar-specific protocol could eliminate any concern about the *Xhv* non-target amplification. HR screening using the BS3272 strain will be necessary to confirm its placement in *Xhv* race 1. However, no other *Xhv* race 1 strains, nor any *Xhv* race 2 strains, were amplified using this primer set. Again, long-read sequencing is expected to provide the resolution needed to confirm the appropriate targets, but these results represent major steps toward *Xhv* race-specific detection.

## 3. Discussion

In previous studies, amplification using the B162 primer pair was used as the initial screening for *Xhv* isolates [52]. The touchdown PCR protocol using the GC3906-152 primer set can now be used for *Xhv* pathovar-specific detection from unknown samples. The specificity of our protocol represents an improvement over previous methods using the B162 primer set [7] designed to detect *Xhv* and LAMP assays [9] designed to detect all members of *X. hortorum* species. In addition to *Xhv* strains, the B162 primer set also amplifies the target 700 bp amplicon in *X. hortorum* pv. *garderi*, *X. hortorum* pv. *cynarae*, *X. hortorum* pv. *taraxaci*, and an *X. hortorum* strain isolated from radicchio. The two pathovars used as the outgroup strains to develop this protocol, *X. hortorum* pv. *carotae* and *X. hortorum* pv. *carotae*, were not amplified in our assays. The LAMP assay as designed by Dia et al. [28] detects an entire clade of *X. hortorum* species, including *Xhv*, *X. hortorum* pv. *gardneri*, and *X. hortorum* pv. *cynarae*.

The *Xhv*-specific primer set described here and the touchdown PCR protocol detected 99%, or 95 out of 96, of the known *Xhv* strains tested (including the pathotype strain CFBP 8686^PT^) and did not detect any of the 33 outgroup strains (demonstrating 100% exclusivity), including 9 closely related *X. hortorum* and *X. hydrangeae* strains and 23 additional *Xanthomonas* type strains. It also successfully detected two *Xhv* isolates from a 2018 bacterial leaf spot outbreak in lettuce in Pennsylvania and did not detect the two *Pseudomonas* isolates from symptomatic tissue from the same outbreak. It is unclear why the reactions with DNA from *Xhv* race 2 BS3126 did not produce the target amplicon using this protocol—this result requires further investigation to determine whether this strain encodes nucleotide variations in the target gene cluster that inhibit primer binding. However, based on its 99% success rate in detecting the collection of 96 known *Xhv* strains and its ability to correctly detect *Xhv* from environmental isolates, we can recommend the use of our touchdown PCR protocol for *Xhv*-specific detection from colony suspensions or DNA extracts. This could replace the less specific conventional PCR method that uses the B162 primers. Further research is necessary to evaluate the efficacy of our protocol for other sample types, such as tissue extracts and seed wash. Additionally, we designed the amplified targets to be within the size range for efficient Taqman RealTime PCR amplification. We are currently evaluating these genomic regions and incorporating additional *Xhv* genomes for the development of a Taqman RealTime PCR for this pathovar. Both its selectivity and sensitivity will be quantified in these assays.

Using the same touchdown PCR method, we evaluated primers designed for *Xhv* race-specific detection; our results appear to highlight the need for long-read sequencing for a variety of *Xhv* strains. The pangenome alignment of our short-read-based assemblies revealed race-specific gene clusters; however, these in silico analyses, along with applying BLAST to search these strains for DNA sequences homologous with these clusters, did not correspond to our laboratory results. GC4021, GC4981, and GC4890 were three gene clusters we identified from the pangenome alignment that were specific to *Xhv* races 1, 2, and 3, respectively. GC4021-112 did not detect any race 2 or 3 strains, but it also only detected 59% of the *Xhv* race 1 strains. GC4981-178 did not detect any race 3 strains, but it did detect 2% (2 out of 82) of the race 1 strains and failed to detect 20% (2 out of 10) of the race 2 strains. GC4980 did not detect any race 2 strains and detected all four race 3 strains, but it also detected 1% (1 out of 82) of the *Xhv* race 1 strains. Despite these drawbacks, we are now using these PCR protocols along with an MLSA to hypothesize about the race of isolates from symptomatic lettuce tissue from novel outbreaks. We hypothesize that our short-read-based sequence assemblies did not capture the full variation that exists in the *Xhv* strain genomes and thus expect that long-read sequences will help to improve our methods. These results also indicate strains for which HR screening will be valuable and may reveal whether any of the strains predicted to belong to a particular race based on MLSA data belong to a different race from that expected. Although the clonal nature of *Xhv* populations suggests that races may correspond to phylogenetic relationships [12,52], the race of a strain is defined by the lettuce cultivar or accession in which it elicits an HR. Differential germplasms for race determination have been described—*L. sativa* cv. Little Gem, *L. serriola* PI491114, and *L. serriola* ARM-09-161-10, for races 1, 2, and 3, respectively [11]—and are being used in subsequent studies.

We expect that our pathovar- and race-specific detection methods will be useful to clinicians, researchers, and seed companies. They will allow for the rapid detection of infection in suspect samples before they result in outbreaks, shown previously to be capable of causing 100% crop loss in severe cases. They may also become useful for seed companies to use to test isolates from seed lots prior to sale or for researchers to use to track the distribution and spread of the pathogen and its races. The race-specific methods, once optimized, could become useful for clinicians who want to provide recommendations to growers for cultivars resistant to the specific *Xhv* race detected from their prior plantings, as the pathogen has been shown to be harbored by weeds and crop debris and cause new outbreaks in subsequent seasons [7].

## 4. Materials and Methods

### 4.1. Isolation, Culturing, and Storage of the Bacterial Strains

Table 2 lists the strains used in this study. The bacterial strains were routinely grown at 28 °C on nutrient agar (NA) and in nutrient broth (NB). Long-term storage was achieved by adding bacteria from pure cultures to 50% nutrient broth and 50% glycerol, vortexing the combination to mix it, and storing it at −80 °C. All of the colony suspensions used in PCRs were created by isolating pure colonies from long-term storage on NA, incubating the plates for three to five days at room temperature, inoculating 30 µL of sterile water in a 1.5 mL Eppendorf tube with a single colony, and vortexing it to mix it.

Following a suspected bacterial leaf spot outbreak in Pennsylvania field-grown lettuce, green leaf and red leaf lettuce samples were submitted to the lab for pathogen isolation and identification. Symptoms appeared as chlorosis and necrotic spots on the leaves, and symptomatic leaves from each lettuce type were used to isolate bacteria using the following procedure. A small tissue fragment was cut out using a sterile scalpel at the margin of a necrotic spot and placed in a sterile 1.5 mL Eppendorf tube. To the tube, 1 mL of 10% sodium hypochlorite was added, and the tube was briefly vortexed. After 1 min of incubation at room temperature, the tube was again briefly vortexed, the sodium hypochlorite was discarded, and the leaf fragment was washed thrice with 1 mL of sterile water. After the final wash was discarded, 30 µL of sterile water was added to the tube, and the tissue was crushed with a sterile pestle. Aliquots (10 µL each) of the resulting plant extract were spread onto two NA plates and incubated for at least two days. Yellow, mucoid, round colonies (typical of *Xanthomonas* strains) that grew were sub-cultured until we achieved purified strains, which were then stored long term at −80 °C in 1.5 mL cryovials that contained 500 µL of sterile NB and 500 µL of sterile glycerol.

### 4.2. Evaluation of the B162 Primer Set

Colony suspensions were prepared as described above for three *Xhv* strains representing the three known races, including *Xhv* BS0347 (race 1), ICMP 1408 (race 2), and BP5181 (race 3), as well as for *Xanthomonas hortorum* pv. *gardneri* (CFBP 8163^PT^), *X. hortorum* pv. *cynarae* (CFBP 4188^PT^), *X. hortorum* pv. *hederae* (CFBP 4925^T^), *X. hortorum* pv. *taraxaci* (CFBP 410^PT^), *X. hortorum* pv. *pelargonii* (CFBP 2533^PT^), *X. hortorum* from radicchio (BP5178), and *X. hortorum* pv. *carotae* (CFBP 7900). These suspensions were used as templates in conventional PCR using the primer set (B162) and the reaction conditions described in Barak et al. [7]. The reactions were run using an MJ Research PTC-100 thermocycler (Montarville, QC, Canada). The PCR products were analyzed using gel electrophoresis; a total of 5 µL of product mixed with 1 µL of loading dye (VWR Life Science EZ-Vision One Dye-as-Loading-Buffer; Radnor, PA, USA) was added to wells of 1% agarose gel, which was then subjected to 84 V for 45 min. Sterile water was loaded in place of the PCR product as the experimental control, and a 1 KB ladder was used as a size reference (New England Biolabs; Ipswich, MA, USA). The amplification bands were visualized using a Bio-Rad Gel Doc XR Imaging System (Hercules, CA, USA).

### 4.3. Genome Alignments and Target Selection

Whole genome sequences for thirteen *Xhv* race 1, four *Xhv* race 2, and two *Xhv* race 2 strains, along with strains from eight closely related *Xanthomonas hortorum* pathovars and one *Xanthomonas hydrangea* strain [12,28], were aligned with a *Xanthomonas hortorum* pv. *carotae* (M081) reference strain via the anvi’o pangenome pipeline [53]. Gene clusters unique to *Xhv* and each of the *Xhv* races were identified and extracted for further analysis. NCBI’s conserved domain tool, BLASTx (database = nr), and BLASTn (database = wgs; organism = ‘*Xanthomonas*’) were used to search for genes with known functions within these clusters and within the B162 amplicon sequence. Matches found in the non-redundant database were considered to be those with at least 90% query coverage, 90% identity, and an e-value threshold of 1 × 10^−5^. The NCBI BLAST versions used in our in silico analyses were 2.12.0 through 2.15.0.

### 4.4. DNA Isolation and Sequencing

Strains from our collection were streaked in quadrants to isolate single colonies, and then individual colonies were used to inoculate 50 µL of sterile NB into 100 mL sterile tubes. The liquid cultures were incubated overnight at 28 °C and shaken at 200 rpm. Genomic DNA was extracted from these cultures using the Qiagen DNeasy UltraClean Microbial Kit (Valencia, CA, USA) according to the manufacturer’s instructions. The DNA concentrations following the final elution were measured using the Thermo Fischer Qubit Fluorometer 3.0 and the Invitrogen dsDNA Broad Range Assay Kit (Waltham, MA, USA). The Genomics Core Facility at the Pennsylvania State University completed all of the Sanger sequencing.

### 4.5. Primer Development

NCBI’s Primer-BLAST was used to design the primers for each of the *Xhv-* or race-specific gene clusters (database = nr; organism = ‘*Xanthomonas*’). Primers were selected from the output that had low or no off-target amplification, low complementarity scores, similar melting temperatures near 60 °C, and a similar GC content near 50%. Custom DNA oligos were ordered from Thermo Fisher Scientific (Waltham, MA, USA). In total, 26 primer sets were evaluated using 19 *Xhv* strains, including strains of all three known races; selected from these primer sets were those that provided the desired pathovar or race specificity (Table 1). All of the other primer sets that were tested are shown in Supplemental Table S2. The detection protocol using the B162 primer set [7] was also evaluated.

### 4.6. Touchdown PCR Development

Initial testing using conventional PCR, involving cycling 34 times under the same conditions as those given in Table 3 but with a consistent annealing temperature of 58 °C during each cycle, did not provide the desired specificity for our detection reactions. Using the touchdown PCR method, using higher annealing temperatures in earlier PCR cycles to reduce non-specific primer binding [54], did provide the appropriate specificity for pathovar- and race-level detection. Each reaction was completed with the following reaction formulation: 9 µL of sterile water, 1.25 µL of each 10 µM primer, 12.5 µL of 2X Bioline Immomix containing IMMOLASE DNA polymerase, and 1 µL of the template, either DNA extract (30 ng/mL) or colony suspension. The colony suspensions were created by taking single colonies from the NA plates, inoculating them into 30 µL of sterile water, and vortexing the combination to mix it. As an experimental control, 1 µL of sterile water was substituted for the template. The reaction conditions are listed in Table 3, and the reactions were run in an MJ Research PTC-100 thermocycler (Montarville, QC, Canada).

Amplified products were separated using gel electrophoresis, as described above in Section 4.2, except that the gels were run with 84 V applied for 2 h given the larger size of these gels. The same 1 KB ladder described above was used as a reference for the amplicon size. The amplification bands were again visualized using the Bio-Rad Gel Doc XR Imaging System (Hercules, CA, USA). A collection of 96 *Xhv* strains, along with the pathotype strains for each of the other *X. hortorum* pathovars, 1 additional *X. hortorum* strain isolated from radicchio [15], and the type strain of *X. hydrangeae* (another close relative of *X. hortorum*), were used to evaluate the specificity of our selected primers. These experiments were conducted twice using colony suspensions from the same strain collection.

### 4.7. The Multilocus Sequence Analysis of the PA Isolates

Single colony suspensions of the strains isolated from the PA lettuce served as the templates for the PCR reactions to separately amplify four *Xanthomonas* housekeeping genes: *rpoD* (693 bp), *fyuA* (762 bp), *gyrB* (522 bp), and *gap1* (774 bp) [10,55]. Bands of the correct sizes were confirmed using gel electrophoresis, the PCR products were cleaned using EXOSAP-IT (Thermo Fisher Scientific, Waltham, MA, USA), and the cleaned products were submitted to the Genetic Core Facility at Penn State University for Sanger sequencing. Using Qiagen’s CLC Genomic Workbench version 21 (Valencia, CA, USA), the forward and reverse sequences for each gene were assembled to the corresponding reference gene sequence of *X. hortorum* pv. *hederae* CFBP 4925^T^. The consensus sequences from each gene were merged into one file for each strain, in the order listed above, and each strain’s merged sequence was aligned with similarly made files but for a collection of *Xhv* strains of known races. The model testing tool, with the default options, was used to determine the appropriate nucleic acid substitution model. As recommended, the general time-reversible model with four categories of rate variation, estimated topology, and 1000 bootstraps was used to generate the maximum likelihood phylogeny.

## Figures and Tables

**Table 1:** Primer sets developed for the specific detection of *Xhv* and its three known races.

Primer Set	Forward Sequence	Reverse Sequence	Amplicon Size (bp)	Target Strains
GC3906-152	GTTCGGTCGCCATTTCGATG	AGATAACCTCCCAGACCGCT	152	*Xhv*
GC4021-112	GGTGGCCTACTTTCATGCGA	GAGCAAGCCCTTCACAAGGT	112	*Xhv* race 1
GC4381-178	TATGATGCGGCACACAACCT	CGTATTGCGGTGCGAACTTT	178	*Xhv* race 2
GC4980-138	TCACTCAAAAGCCCACCCTC	ACATTCCTCGGCTATCCCCT	138	*Xhv* race 3

**Table 2:** A list of the bacterial strains that were included in this study.

Organism	Strain *	Other Strain IDs	Host of Isolation	Known or Hypothesized Race	GC3906-152 Detection	GC4021-112 Detection	GC4381-178 Detection	GC4980-138 Detection	Origin	Collector or Citation
*X. hortorum* pv. *vitians*	**BP5172** **‡**	*Xav* 98-37 2/01	*Lactuca sativa*	*Xhv* race 1	Yes	No	No	No	Salinas, CA, USA	J. Barak
*X. hortorum* pv. *vitians*	**BS0339 ‡**	Salinas 2/01	*Lactuca sativa*	*Xhv* race 1	Yes	Yes	No	No	Salinas, CA, USA	J. Barak
*X. hortorum* pv. *vitians*	**BS0340 ‡**	*Xav* 98-23 2/01	*Lactuca sativa*	*Xhv* race 1	Yes	No	No	No	Salinas, CA, USA	J. Barak
*X. hortorum* pv. *vitians*	**BS0347 ‡**	*Xcv* 5/01	*Lactuca sativa*	*Xhv* race 1	Yes	Faint	No	No	Salinas, CA, USA	J. Barak
*X. hortorum* pv. *vitians*	**BP5176 ‡**	*Xcv* 5/01	*Lactuca sativa*	*Xhv* race 1	Yes	Yes	No	No	Salinas, CA, USA	J. Barak
*X. hortorum* pv. *vitians*	**BP5177 ‡**	“Edge A”	*Lactuca sativa*	*Xhv* race 1	Yes	No	No	No	CO, USA	S. Koike
*X. hortorum* pv. *vitians*	**BP5179 ‡**	“Daniel Rom”	*Lactuca sativa*	*Xhv* race 1	Yes	Yes	No	No	Salinas, CA, USA	J. Barak
*X. hortorum* pv. *vitians*	**BP5182 ‡**	“Moreno Let”	*Lactuca sativa*	*Xhv* race 1	Yes	Yes	Yes	No	Santa Maria, CA, USA	J. Barak
*X. hortorum* pv. *vitians*	**NCPPB 4058 ‡**	N/A	*Lactuca sativa*	*Xhv* race 1	Yes	Yes	No	No	UK	H. Stanford
*X. hortorum* pv. *vitians*	**CFBP 8686^PT^ ‡**	LMG 938^PT^, NCPPB 2248^PT^, MR20213^PT^	*Lactuca sativa*	*Xhv* race 1	Yes	Yes	No	No	Zimbabwe	[6,8]
*X. hortorum* pv. *vitians*	**BP5191 ‡**	VT111	*Lactuca sativa*	*Xhv* race 1	Yes	No	No	No	Canada	V. Toussaint
*X. hortorum* pv. *vitians*	**BP5192 ‡**	*Xcv*-2	*Lactuca sativa*	*Xhv* race 1	Yes	Yes	No	No	CA, USA	C. T. Bull
*X. hortorum* pv. *vitians*	**ICMP 1408 ‡**	PDDCC 1408, Burkholder XL5	*Lactuca sativa*	*Xhv* race 2	Yes	No	Yes	No	Ithaca, NY, USA	W. H. Burkholder
*X. hortorum* pv. *vitians*	**ICMP 4165 ‡**	LMG 7508, PDDCC 4165	*Lactuca sativa*	*Xhv* race 2	Yes	No	Yes	No	New Zealand	H. J. Boesewinkel
*X. hortorum* pv. *vitians*	**BS3127 ‡**	VT106	*Lactuca sativa*	*Xhv* race 2	Yes	No	Yes	No	Canada	V. Toussaint
*X. hortorum* pv. *vitians*	**BP5194 ‡**	917	*Lactuca sativa*	*Xhv* race 2	Yes	No	Yes	No	OH, USA	[18]
*X. hortorum* pv. *vitians*	**BS2861 ‡**	“Christy BuLet 2”	*Lactuca sativa*	*Xhv* race 3	Yes	No	No	Yes	King City, CA, USA	S. Koike and Rianda
*X. hortorum* pv. *vitians*	**BP5181 ‡**	“Christy BuLet 3”	*Lactuca sativa*	*Xhv* race 3	Yes	No	No	Yes	King City, CA, USA	S. Koike and Rianda
*X. hortorum* pv. *vitians*	BS0313	A674-2B (e1)	*Lactuca sativa*	Hypothesized *Xhv* race 1	Yes	Yes	No	No	HI, USA	A. Alvarez
*X. hortorum* pv. *vitians*	BS0341	C 5/20/01	*Lactuca sativa*	Hypothesized *Xhv* race 1	Yes	Yes	No	No	Salinas, CA, USA	J. Barak
*X. hortorum* pv. *vitians*	BS0342	*Xcv* 5/20/01	*Lactuca sativa*	Hypothesized *Xhv* race 1	Yes	Yes	No	No	Salinas, CA, USA	J. Barak
*X. hortorum* pv. *vitians*	BS0343	*Xcv* 5/01	*Lactuca sativa*	Hypothesized *Xhv* race 1	Yes	Yes	No	No	Salinas, CA, USA	J. Barak
*X. hortorum* pv. *vitians*	BS0345	*Xcv* 5/01	*Lactuca sativa*	Hypothesized *Xhv* race 1	Yes	Yes	No	No	Salinas, CA, USA	J. Barak
*X. hortorum* pv. *vitians*	BS0346	*Xcv* 5/01	*Lactuca sativa*	Hypothesized *Xhv* race 1	Yes	Yes	No	No	Salinas, CA, USA	J. Barak
*X. hortorum* pv. *vitians*	BS2849	“Mike Lombard Reeves”	*Lactuca sativa*	Hypothesized *Xhv* race 1	Yes	Yes	No	No	Salinas, CA, USA	S. Koike
*X. hortorum* pv. *vitians*	BS2850	“Mike Lombard Harden”	*Lactuca sativa*	Hypothesized *Xhv* race 1	Yes	Yes	No	No	Salinas, CA, USA	S. Koike
*X. hortorum* pv. *vitians*	BS2852	“Daniel Rom”	*Lactuca sativa*	Hypothesized *Xhv* race 1	Yes	Yes	No	No	Salinas, CA, USA	S. Koike
*X. hortorum* pv. *vitians*	BS2855	“Frank Let”	*Lactuca sativa*	Hypothesized *Xhv* race 1	Yes	Yes	No	No	Salinas, CA, USA	S. Koike
*X. hortorum* pv. *vitians*	BS2857	“Frank Let”	*Lactuca sativa*	Hypothesized *Xhv* race 1	Yes	Yes	No	No	Salinas, CA, USA	S. Koike
*X. hortorum* pv. *vitians*	BS2858	John DeCarli Rom1-1	*Lactuca sativa*	Hypothesized *Xhv* race 1	Yes	Yes	No	No	Salinas, CA, USA	S. Koike
*X. hortorum* pv. *vitians*	BS2859	John DeCarli Rom1-2	*Lactuca sativa*	Hypothesized *Xhv* race 1	Yes	Yes	No	No	Salinas, CA, USA	S. Koike
*X. hortorum* pv. *vitians*	BS2870	“Moreno Let”	*Lactuca sativa*	Hypothesized *Xhv* race 1	Yes	Yes	No	No	Santa maria, CA, USA	S. Koike
*X. hortorum* pv. *vitians*	BS2871	“Keller Rom”	*Lactuca sativa*	Hypothesized *Xhv* race 1	Yes	Yes	No	No	Salinas, CA, USA	S. Koike
*X. hortorum* pv. *vitians*	BS2872	“Keller Rom”	*Lactuca sativa*	Hypothesized *Xhv* race 1	Yes	Yes	No	No	Salinas, CA, USA	S. Koike
*X. hortorum* pv. *vitians*	BS2873	“Greg greenleaf”	*Lactuca sativa*	Hypothesized *Xhv* race 1	Yes	Yes	No	No	Salinas, CA, USA	S. Koike
*X. hortorum* pv. *vitians*	BS2874	“Greg greenleaf”	*Lactuca sativa*	Hypothesized *Xhv* race 1	Yes	Yes	No	No	Salinas, CA, USA	S. Koike
*X. hortorum* pv. *vitians*	BS2875	“Greg redleaf”	*Lactuca sativa*	Hypothesized *Xhv* race 1	Yes	Yes	No	No	Salinas, CA, USA	S. Koike
*X. hortorum* pv. *vitians*	BS2876	“Greg let”	*Lactuca sativa*	Hypothesized *Xhv* race 1	Yes	Yes	No	No	Salinas, CA, USA	S. Koike
*X. hortorum* pv. *vitians*	BS2908	“Matt romaine”	*Lactuca sativa*	Hypothesized *Xhv* race 1	Yes	Yes	No	No	Watsonville, CA, USA	S. Koike
*X. hortorum* pv. *vitians*	BS2994	ICMP 6656, DAR 30547	*Lactuca sativa*	Hypothesized *Xhv* race 1	Yes	Yes	No	No	New South Wales, Australia	R. Fitzell
*X. hortorum* pv. *vitians*	BS2996	ICMP 6735, Watson B2578	*Lactuca sativa*	Hypothesized *Xhv* race 1	Yes	Yes	Yes	No	Palmerston North, WI, New Zealand	D. R. W. Watson
*X. hortorum* pv. *vitians*	BS2997	ICMP 7423, Watson J2928.	*Lactuca sativa*	Hypothesized *Xhv* race 1	Yes	Yes	No	No	Patumahoe, AK, New Zealand	D. R. W. Watson
*X. hortorum* pv. *vitians*	BS3050	LMG 8688; ICMP 6461; Watson D2538	*Lactuca sativa*	Hypothesized *Xhv* race 1	Yes	No	No	No	New Zealand	D. R. W. Watson
*X. hortorum* pv. *vitians*	BS3051	LMG 8690; Hill AS3997; ICMP 6857	*Lactuca sativa*	Hypothesized *Xhv* race 1	Yes	Yes	No	No	New Zealand	D. R. W. Watson
*X. hortorum* pv. *vitians*	BS3054	CFBP 3980, Audusseau 11.72	*Lactuca sativa*	Hypothesized *Xhv* race 1	Yes	No	No	No	Vaucluse, France	C. Audusseau
*X. hortorum* pv. *vitians*	BS3056	CFBP 3996 Audusseau 17.09	*Lactuca sativa*	Hypothesized *Xhv* race 1	Yes	Yes	No	No	Isère, France	C. Audusseau
*X. hortorum* pv. *vitians*	BS3128	105 VT107	*Lactuca sativa*	Hypothesized *Xhv* race 1	Yes	Yes	No	No	Canada	V. Toussaint
*X. hortorum* pv. *vitians*	BS3131	119 VT41	*Lactuca sativa*	Hypothesized *Xhv* race 1	Yes	Yes	No	No	Canada	V. Toussaint
*X. hortorum* pv. *vitians*	BS3132‡	B07-007, ID200707A	*Lactuca sativa*	*Xhv* race 1	Yes	Yes	No	No	Canada	V. Toussaint
*X. hortorum* pv. *vitians*	BS3272	L11	*Lactuca sativa*	Hypothesized *Xhv* race 1	Yes	Yes	No	Yes	FL, USA	[2]
*X. hortorum* pv. *vitians*	BS3300	*Xcv*-4	*Lactuca sativa*	Hypothesized *Xhv* race 1	Yes	Yes	No	No	CA, USA	C. T. Bull
*X. hortorum* pv. *vitians*	BS3301	*Xcv*-5	*Lactuca sativa*	Hypothesized *Xhv* race 1	Yes	Yes	No	No	CA, USA	C. T. Bull
*X. hortorum* pv. *vitians*	BS3303	*Xcv*-7	*Lactuca sativa*	Hypothesized *Xhv* race 1	Yes	Yes	No	No	CA, USA	C. T. Bull
*X. hortorum* pv. *vitians*	BS3304	*Xcv*-8	*Lactuca sativa*	Hypothesized *Xhv* race 1	Yes	Yes	No	No	CA, USA	C. T. Bull
*X. hortorum* pv. *vitians*	BS3306	*Xcv*-10	*Lactuca sativa*	Hypothesized *Xhv* race 1	Yes	Yes	No	No	CA, USA	C. T. Bull
*X. hortorum* pv. *vitians*	BS0301	10S7-2 (a2)	*Lactuca sativa*	Hypothesized *Xhv* race 1	Yes	No	No	No	HI, USA	A. Alvarez
*X. hortorum* pv. *vitians*	BS0302	10TB7 (a4)	*Lactuca sativa*	Hypothesized *Xhv* race 1	Yes	No	No	No	HI, USA	A. Alvarez
*X. hortorum* pv. *vitians*	BS0304	10TB9 (a6)	*Lactuca sativa*	Hypothesized *Xhv* race 1	Yes	No	No	No	HI, USA	A. Alvarez
*X. hortorum* pv. *vitians*	BS0305	10TB12-1 (a7)	*Lactuca sativa*	Hypothesized *Xhv* race 1	Yes	No	No	No	HI, USA	A. Alvarez
*X. hortorum* pv. *vitians*	BS0309	S4-1(K) (c5)	*Lactuca sativa*	Hypothesized *Xhv* race 1	Yes	No	No	No	HI, USA	A. Alvarez
*X. hortorum* pv. *vitians*	BS0310	S5-2 (c6)	*Lactuca sativa*	Hypothesized *Xhv* race 1	Yes	Faint	No	No	HI, USA	A. Alvarez
*X. hortorum* pv. *vitians*	BS0311	S5-2(K) (c7)	*Lactuca sativa*	Hypothesized *Xhv* race 1	Yes	No	No	No	HI, USA	A. Alvarez
*X. hortorum* pv. *vitians*	BS0314	A674-4B (e2)	*Lactuca sativa*	Hypothesized *Xhv* race 1	Yes	No	No	No	HI, USA	A. Alvarez
*X. hortorum* pv. *vitians*	BS0316	10TB9 (e4)	*Lactuca sativa*	Hypothesized *Xhv* race 1	Yes	No	No	No	HI, USA	A. Alvarez
*X. hortorum* pv. *vitians*	BS0318	QR71B (e8)	*Lactuca sativa*	Hypothesized *Xhv* race 1	Yes	Yes	No	No	HI, USA	A. Alvarez
*X. hortorum* pv. *vitians*	BS0335	*Xav* 98-05 2/01	*Lactuca sativa*	Hypothesized *Xhv* race 1	Yes	No	No	No	Salinas, CA, USA	J. Barak
*X. hortorum* pv. *vitians*	BS0337	*Xav* 98-67 2/01	*Lactuca sativa*	Hypothesized *Xhv* race 1	Yes	Yes	No	No	Salinas, CA, USA	J. Barak
*X. hortorum* pv. *vitians*	BS0338	*Xav* 98-76 2/01	*Lactuca sativa*	Hypothesized *Xhv* race 1	Yes	Yes	No	No	Salinas, CA, USA	J. Barak
*X. hortorum* pv. *vitians*	BS0542	10S7-2 (a2)	*Lactuca sativa*	Hypothesized *Xhv* race 1	Yes	No	No	No	Salinas, CA, USA	J. Barak
*X. hortorum* pv. *vitians*	BS0543	“Spot A”	*Lactuca sativa*	Hypothesized *Xhv* race 1	Yes	No	No	No	Salinas, CA, USA	J. Barak
*X. hortorum* pv. *vitians*	BS2946	Julio Rodrigues Neto IBSBF 1553 Embrapa K 532	*Lactuca sativa*	Hypothesized *Xhv* race 1	Yes	No	No	No	Brazil	I. M. G. Almeida
*X. hortorum* pv. *vitians*	BS2998	ICMP 7465, IBSBF 325, C.F. Robbs: ENA2008	*Lactuca sativa*	Hypothesized *Xhv* race 1	Yes	No	No	No	Brazil	C. F. Robbs
*X. hortorum* pv. *vitians*	BS3035	NCPPB 970, ICPB XL 102, Thornberry 1-49	*Lactuca sativa*	Hypothesized *Xhv* race 1	Yes	No	No	No	USA	H. H. Thornberry
*X. hortorum* pv. *vitians*	BS3036	NCPPB 1839, ICPB XV169, Robbs ENA-250	*Lactuca sativa*	Hypothesized *Xhv* race 1	Yes	No	No	No	Brazil	A. P. Viegas
*X. hortorum* pv. *vitians*	BS3041	NCPPB 3663, Neto ISBF 473	*Lactuca sativa*	Hypothesized *Xhv* race 1	Yes	No	No	No		J. R. Neto
*X. hortorum* pv. *vitians*	BS3042	NCPPB3931, Sellwood/Wilson A6520/1	*Lactuca sativa*	Hypothesized *Xhv* race 1	Yes	No	No	No	The UK	J.E. Sellwood and J.K. Wilson
*X. hortorum* pv. *vitians*	BS3047	LMG 7453; ATCC 11525; Burkholder XL3; CNBP 500; Dye YA3; ICMP 337; ICPB XL3; NCPPB 992; PDDCC 337	*Lactuca sativa*	Hypothesized *Xhv* race 1	Yes	No	No	No	USA	H. Anderson
*X. hortorum* pv. *vitians*	BS3049	LMG 7510; Fahy DAR30526; ICMP 6655; PDDCC 6655	*Lactuca sativa*	Hypothesized *Xhv* race 1	Yes	No	No	No	Australia	P. Fahy
*X. hortorum* pv. *vitians*	BS3053	CFBP 3973, Audusseau 11.08	*Lactuca sativa*	Hypothesized *Xhv* race 1	Yes	No	No	No		C. Audusseau
*X. hortorum* pv. *vitians*	BS3055	CFBP 3983, Audusseau 14.28	*Lactuca sativa*	Hypothesized *Xhv* race 1	Yes	No	No	No	Isère, France	C. Audusseau
*X. hortorum* pv. *vitians*	BS3130	118 VT25	*Lactuca sativa*	Hypothesized *Xhv* race 1	Yes	No	No	No		V. Toussaint
*X. hortorum* pv. *vitians*	BS3271	L43	*Lactuca sativa*	Hypothesized *Xhv* race 1	Yes	No	No	No	FL, USA	C. T. Bull
*X. hortorum* pv. *vitians*	BS3526	B55	*Lactuca sativa*	Hypothesized *Xhv* race 1	Yes	No	No	No	CA, USA	[18]
*X. hortorum* pv. *vitians*	BS3527	B57	*Lactuca sativa*	Hypothesized *Xhv* race 1	Yes	No	No	No	CA, USA	[18]
*X. hortorum* pv. *vitians*	BS0344 ‡	*Xcv* 5/01	*Lactuca sativa*	*Xhv* race 1	Yes	Yes	No	No	Salinas, CA, USA	J. Barak
*X. hortorum* pv. *vitians*	BS2851 ‡	“Mike Lombard Harden”	*Lactuca sativa*	*Xhv* race 1	Yes	Yes	No	No	Salinas, CA, USA	S. Koike
*X. hortorum* pv. *vitians*	BS2909	“Matt romaine”	*Lactuca sativa*	Hypothesized *Xhv* race 1	Yes	Yes	No	No	Watsonville, CA, USA	S. Koike
*X. hortorum* pv. *vitians*	BS3528 ‡	B59	*Lactuca sativa*	*Xhv* race 1	Yes	Yes	No	No	CA, USA	[18]
*X. hortorum* pv. *vitians*	BS3034 ‡	NCPPB 2969, ICPB XL6	*Lactuca sativa*	*Xhv* race 2	Yes	No	Yes	No	USA	W. H. Burkholder
*X. hortorum* pv. *vitians*	BS3043 ‡	NCPPB 4033, Sahin/Miller 700a	*Lactuca sativa*	*Xhv* race 2	Yes	No	Yes	No	USA	F. Sahin
*X. hortorum* pv. *vitians*	BS3126 ‡	99 VT101	*Lactuca sativa*	*Xhv* race 2	No	No	Yes	No	Isère, France	C. Audusseau
*X. hortorum* pv. *vitians*	BS3529 ‡	906	*Lactuca sativa*	*Xhv* race 2	Yes	No	Yes	No	OH, USA	S. Miller
*X. hortorum* pv. *vitians*	BS3531 ‡	923	*Lactuca sativa*	*Xhv* race 2	Yes	No	No	No	OH, USA	[18]
*X. hortorum* pv. *vitians*	BS3532 ‡	924	*Lactuca sativa*	*Xhv* race 2	Yes	No	No	No	OH, USA	[18]
*X. hortorum* pv. *vitians*	BS2860	Christy BuLet 1	*Lactuca sativa*	Hypothesized *Xhv* race 3	Yes	No	No	Yes	King City, CA, USA	S. Koike
*X. hortorum* pv. *vitians*	BS2863	Christy BuLet 4	*Lactuca sativa*	Hypothesized *Xhv* race 3	Yes	No	No	Yes	King City, CA, USA	S. Koike
*X. hortorum* pv. *vitians*	BP4476	N/A	*Lactuca sativa*	Hypothesized *Xhv* race 1	Yes	Not tested	Not tested	Not tested	PA, USA	This study
*X. hortorum* pv. *vitians*	BP4477	N/A	*Lactuca sativa*	Hypothesized *Xhv* race 1	Yes	Not tested	Not tested	Not tested	PA, USA	This study
*Pseudomonas viridiflava*	BP4478	N/A	*Lactuca sativa*	N/A	No	Not tested	Not tested	Not tested	PA, USA	This study
*Pseudomonas allivorans*	BP4479	N/A	*Lactuca sativa*	N/A	No	Not tested	Not tested	Not tested	PA, USA	This study
*X. hortorum*	**BP5178**	N/A	*Cichorium intybus* (radicchio)	N/A	No	No	No	No	Salinas, CA, USA	[17]
*X. hortorum* pv. hederae	**CFBP 4925^T^**	ICMP 453^T^, NCPPB 939^T^, LMG 733^T^	*Hedera helix* (English ivy)	N/A	No	No	No	No	USA	[8,19,20]
*X. hortorum* pv. taraxaci	**CFBP 410^PT^**	ATCC 19318^PT^, NCPPB 940^PT^, LMG 870^PT^	*Taraxacum kok-sahgyz* (Russian dandelion)	N/A	No	No	No	No	Ithaca, NY, USA	[8,19,20,21]
*X. hortorum* pv. pelargonii	**CFBP 2533^PT^**	ICMP 4321PT, LMG 7314^PT^, NCPPB 2985^PT^	*Pelargonium peltatum* (pelargonium)	N/A	No	No	No	No	Auckland, New Zealand	[20,22]
*X. hortorum* pv. gardneri	**CFBP 8163^PT^**	LMG 962^PT^, ATCC19865^PT^, NCPPB 881^PT^, PDCC 1620^PT^	*Solanum lycopersicum* (tomato)	N/A	No	No	No	No	Yugoslavia	[23,24]
*X. hortorum* pv. cynarae	**CFBP 4188^PT^**	ICMP 16775^PT^	*Cynara scolymus* (artichoke)	N/A	No	No	No	No	France	[25]
*X. hortorum* pv. carotae	**CFBP 7900**	M081	*Daucus carota* (carrot)	N/A	No	No	No	Faint	Hungary	[8,20,26]
*X. campestris* pv. coriandri	**CFBP 8452^PT^**	LMG 687^PT^, ATCC 17996^PT^, ICMP 5725^PT^, NCPPB 1758^PT^, PDDCC 5725^PT^	*Coriandrum sativum* (coriander)	N/A	No	No	No	No	India	[8,20,27]
*X. hydrangeae*	**LMG 31884^T^**	Van Vaerenbergh gbbc963^T^; GBBC 2123^T^, CCOS 1956^T^	*Hydrangea arborescens* (smooth hydrangea)	N/A	No	Not tested	Not tested	Not tested	Flanders, Belgium	[28]
*X. axonopodis*	CFBP 4924^T^	ATCC 19312^T^, CFBP 2156^T^, ICMP 50^T^, ICPB Xa103^T^, LMG 538^T^, LMG 982^T^, NCPPB 457^T^	*Axonopus scoparius* (carpet grass)	N/A	No	Not tested	Not tested	Not tested	Colombia	[29]
*X. campestris*	CFBP 5251^T^	ATCC 33913^T^, CFBP 2350^T^, CFBP 5241^T^, DSM 3586^T^, ICMP 13^T^, LMG 568^T^, Labo 11405^T^, NCPPB 528^T^	*Brassica oleracea var. gemmifera* (Brussels sprout)	N/A	No	Not tested	Not tested	Not tested	The UK	[30,31]
*X. translucens*	CFBP 2054^T^	ATCC 19319^T^, ICMP 5752^T^, ICPB XT2^T^, LMG 876^T^, NCPPB 973^T^	*Hordeum vulgare* (barley)	N/A	No	Not tested	Not tested	Not tested	USA	[8,32]
*X. theicola*	CFBP 4691^T^	ICMP 6774^T^, LMG 8684^T^	*Camellia sinensis* (tea tree)	N/A	No	Not tested	Not tested	Not tested	Japan	[8]
*X. arboricola*	CFBP 2528^T^	ATCC 49083^T^, ICMP 35^T^, LMG 747^T^, NCPPB 411^T^	*Juglans regia* (walnut)	N/A	No	Not tested	Not tested	Not tested	New Zealand	[8,33]
*X. cucurbitae*	CFBP 2542^T^	ICMP 2299^T^, LMG 690^T^, NCPPB 2597^T^	*Cucurbita maxima* (squash)	N/A	No	Not tested	Not tested	Not tested	New Zealand	[8,34]
*X. alfalfae*	CFBP 7686^T^	ATCC 11765^T^, ICPB 10701^T^, ICPB XA 121^T^, LMG 495^T^	*Medicago sativa* (alfalfa)	N/A	No	Not tested	Not tested	Not tested	India	[35,36]
*X. sacchari*	CFBP 4641^T^	LMG 471^T^	*Saccharum officinarum* (sugarcane)	N/A	No	Not tested	Not tested	Not tested	Guadeloupe, France	[8]
*X. melonis*	CFBP 4644^T^	IBSBF 68^T^, ICMP 8682^T^, LMG 8670^T^, NCPPB 3434^T^	*Cucumis melo* (melon)	N/A	No	Not tested	Not tested	Not tested	Brazil	[8]
*X. hyacinthi*	CFBP 1156^T^	ATCC 19314^T,^ ICMP 189^T^, LMG 739^T^, NCPPB 599^T^	*Hyacinthus orientalis* (hyacinth)	N/A	No	Not tested	Not tested	Not tested	Netherlands	[8,37]
*X. phaseoli*	CFBP 8462^T^	ATCC 49119^T^, LMG 29033^T^	*Phaseolus vulgaris* (common bean)	N/A	No	Not tested	Not tested	Not tested	NE, USA	[38,39]
*X. bromi*	CFBP 1976^T^	ICMP 12545^T^, LMG 947^T^	*Bromus carinatus* (bromegrass)	N/A	No	Not tested	Not tested	Not tested	France	[8]
*X. euvesicatoria*	CFBP 6864^T^	ATCC 11633^T^, DSM 19128^T^, ICMP 109^T^, ICMP 98^T^, NCPPB 2968^T^	*Capsicum frutescens* (wild chili pepper)	N/A	No	Not tested	Not tested	Not tested	USA	[23]
*X. vasicola*	CFBP 2543^T^	ICMP 3103^T^, LMG 736^T^, NCPPB 2417^T^	*Sorghum vulgare* (sorghum)	N/A	No	Not tested	Not tested	Not tested	New Zealand	[8,40]
*X. cassavae*	CFBP 4642^T^	ICMP 204^T^, LMG 673^T^, NCPPB 101^T^	*Manihot esculenta* (cassava)	N/A	No	Not tested	Not tested	Not tested	Malawi	[8,41]
*X. populi*	CFBP 1817^T^	ATCC 51165^T^, ICMP 5816^T^, ICPB XP 240^T^, LMG 5743^T^	*Populus x canadensis cv. Regenerata* (Canadian poplar)	N/A	No	Not tested	Not tested	Not tested	Noyon, Oise, France	[42,43]
*X. vesicatoria*	CFBP 2537^T^	ATCC 35937^T^, CFBP 4645^T^, ICMP 63^T^, LMG 911^T^, NCPPB 422^T^	*Lycopersicon esculentum* (tomato)	N/A	No	Not tested	Not tested	Not tested	New Zealand	[8,44]
*X. pisi*	CFBP 4643^T^	ATCC 35936^T^, ICMP 570^T^, ICMP 570^T^, LMG 847^T^, Labo 13356^T^, NCPPB 762^T^	*Pisum sativum* (pea)	N/A	No	Not tested	Not tested	Not tested	Japan	[8,45]
*X. dyei*	CFBP 7245^T^	DSM 110537^T^, ICMP 12167^T^, NCPPB 4446^T^	*Metrosideros excelsa* (New Zealand Christmas tree)	N/A	No	Not tested	Not tested	Not tested	Omahanui, Bay of Plenty, New Zealand	[46]
*X. citri*	CFBP 3369^T^	ATCC 49118^T^, LMG 9322^T^	*Citrus aurantifolia* (key lime)	N/A	No	Not tested	Not tested	Not tested	FL, USA	[38,47,48]
*X. albilineans*	CFBP 2523^T^	ATCC 33915^T^, ICMP 196^T^, LMG 494^T^, NCPPB 2969^T^	*Saccharum officinarum* (sugarcane)	N/A	No	Not tested	Not tested	Not tested	Fiji	[49,50,51]
*X. perforans*	CFBP 7293^T^	DSM 18975^T^, NCPPB 4321^T^	*Solanum lycopersicum L.* (tomato)	N/A	No	Not tested	Not tested	Not tested	FL, USA	[23]
*X. codiae*	CFBP 4690^T^	ICMP 9513^T^, LMG 8678^T^	*Codiacum variegatum* (croton)	N/A	No	Not tested	Not tested	Not tested	FL, USA	[8]

* Strains in bold were used in the whole genome sequence alignments to identify *Xhv*-specific and race-specific gene cluster targets for PCR-based detection. *Xhv* strains marked with ‡ have had their race confirmed via HR screening [10,11], while those lacking the ‡ symbol have been hypothesized to belong to their race based on a multilocus sequence analysis (MLSA).

**Table 3:** Touchdown PCR cycling conditions.

Step	Instruction	Purpose
1	95 °C for 1 min	Taq polymerase activation
2 *	95 °C for 30 s	Denaturation
3 *	68 °C for 30 s, −1 °C every cycle	Annealing
4 *	72 °C for 30 s	Extension
5 *	GOTO Step 2, 10 times	Cycling
6	95 °C for 30 s	Denaturation
7	58 °C for 30 s	Annealing
8	72 °C for 30 s	Extension
9	GOTO Step 6, 23 times	Cycling
10	72 °C for 5 min	Final extension

* The touchdown phase of our protocol was when the annealing temperature was kept higher than that in typical PCR for 11 cycles to reduce non-specific primer binding. The remaining cycling temperatures were typical of general PCR.

## Data Availability

The original contributions presented in this study are included in the article/Supplementary Materials. Further inquiries can be directed to the corresponding author.

## Supplementary Materials

The following supporting information can be downloaded at: https://www.mdpi.com/article/10.3390/plants14060964/s1. Figure S1: BLASTx protein alignment for top hit (A) and BLASTn DNA alignments (B) to query B162 amplicon sequence. Table S1: The gene clusters identified using anvi’o and the in silico evaluation of their content and specificity. Figure S2: BLASTx protein alignments for top hits (A) and BLASTn DNA alignments (B) to query GC3906 sequence. Figure S3: BLASTx protein alignments for top hits (A) and BLASTn DNA alignments (B) to query GC4021 sequence. Figure S4: BLASTx protein alignments for top hits (A) and BLASTn DNA alignments (B) to query GC4381 sequence. Figure S5: BLASTx protein alignments for top hits (A) and BLASTn DNA alignments (B) to query GC4980 sequence. Figure S6: *Xhv*-specific detection using the touchdown PCR method and the GC3906-152 primer set. Figure S7: Partial *Xhv* race 1 detection using the touchdown PCR method and the GC4021-112 primer set. Figure S8: *Xhv* race 2 detection using the touchdown PCR method and the GC4381-178 primer set. Figure S9: *Xhv* race 3 detection using the touchdown PCR method and the GC4980-138 primer set. Table S2: Additional primers developed and tested for specificity to potential *Xhv* pathovar- or race-specific gene cluster targets.

## Abbreviations

The following abbreviations are used in this manuscript:

*Xhv*	*Xanthomonas hortorum* pv. *vitians*
LAMP	Loop-mediated isothermal amplification
HR	Hypersensitive response
SNP	Single-nucleotide polymorphism
PCR	Polymerase chain reaction
DNA	Deoxyribonucleic acid
bp	Base pair
KB	Kilobase
NCBI	National Center for Biotechnology Information
BLAST	Basic local alignment search tool
ANI	Average nucleotide identity
gDDH	Genome-based DNA-DNA hybridization
NEB	New England Biolabs
MLSA	Multilocus sequence analysis
