# Supplementary material for: Novel PCR-Based Detection Methods for the Lettuce Bacterial Leaf Spot Pathogen, Xanthomonas hortorum pv. vitians Morinière et al., 2020
Source: Plants (Basel). 2025 Mar 19;14(6):964. doi: 10.3390/plants14060964 (PMC11944428; doi:10.3390/plants14060964)
Supplement: Supplementary file 1 [file plants-14-00964-s001.zip › plants-3469597-supplementary.pdf]

Martinez et al., 2025; Supplemental Figures 1-9

A **glycosyl hydrolase family 3, partial [Xanthomonas campestris pv. vitians]**

Sequence ID: AXG24216.1 Length: 220 Number of Matches: 1

See 1 more title(s) See all Identical Proteins(IPG)

Range 1: 6 to 216 GenPept Graphics ▼ Next Match ▲ Previous Match

Score	Expect	Method	Identities	Positives	Gaps	Frame
434 bits(1115)	3e-153	Compositional matrix adjust.	211/211(100%)	211/211(100%)	0/211(0%)	-3

```
Query  633  SNHVGGVILFSNNIKERSQIEKLTSWYAGMESSAGVHLLIATDNEGGNVFRLPRNEYASF  454
            SNHVGGVILFSNNIKERSQIEKLTSWYAGMESSAGVHLLIATDNEGGNVFRLPRNEYASF
Sbjct  6    SNHVGGVILFSNNIKERSQIEKLTSWYAGMESSAGVHLLIATDNEGGNVFRLPRNEYASF  65

Query  453  PGNMALAAAIEGGSSEQLAFEQGRLLAQDLLALKINTNFAPVADVNANPFNPVINVRAFS  274
            PGNMALAAAIEGGSSEQLAFEQGRLLAQDLLALKINTNFAPVADVNANPFNPVINVRAFS
Sbjct  66   PGNMALAAAIEGGSSEQLAFEQGRLLAQDLLALKINTNFAPVADVNANPFNPVINVRAFS  125

Query  273  DNADVVSRLAGKIAAGMERQGLVTTYKHFPGHGSTSTDSHTGLPRVDLSRDQAFAIDIAP  94
            DNADVVSRLAGKIAAGMERQGLVTTYKHFPGHGSTSTDSHTGLPRVDLSRDQAFAIDIAP
Sbjct  126  DNADVVSRLAGKIAAGMERQGLVTTYKHFPGHGSTSTDSHTGLPRVDLSRDQAFAIDIAP  185

Query  93   YQQAISAHAAPDMVMTAHIQYPALDETLGTN  1
            YQQAISAHAAPDMVMTAHIQYPALDETLGTN
Sbjct  186  YQQAISAHAAPDMVMTAHIQYPALDETLGTN  216
```

B

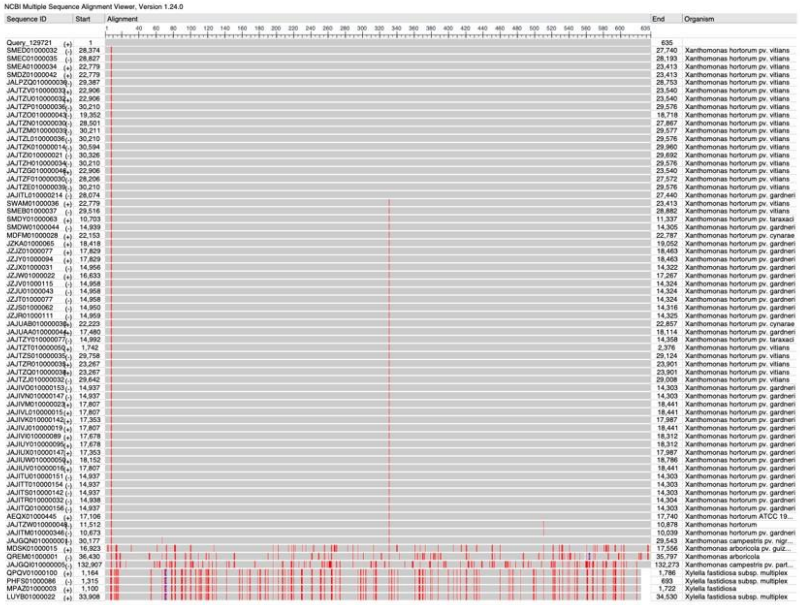

Figure S1. BLASTx protein alignment for top hit (A) and BLASTn DNA alignments (B) to query B162 amplicon sequence. (A) The top protein match to the B162 amplicon DNA sequence query, an *Xhv* (formerly *X. campestris* pv. *vitians*) glycosyl hydrolase family 3 enzyme, with 100% identity **(B)** Multiple sequence alignment of the B162 amplicon DNA sequence query to top DNA hits. Red bars represent nucleotide substitutions, white bars represent gaps, and blue 'I' symbols represent insertions. Whole genomes of *Xhv* strains submitted by Rosenthal et al., 2022 include those with sequence IDs starting with 'JAJTZ.'

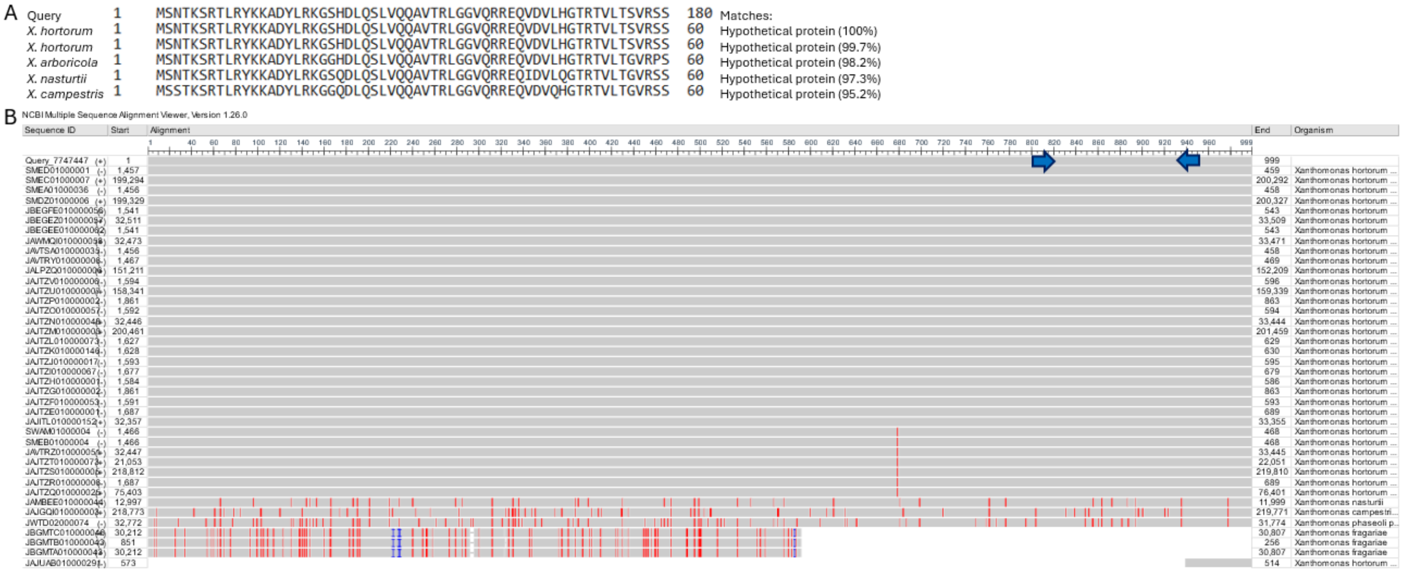

Figure S2. BLASTx protein alignments for top hits (A) and BLASTn DNA alignments (B) to query GC3906 sequence. (A) The top five protein matches to the GC3906 query DNA sequence; percent identity value for each match is shown in parentheses. **(B)** Multiple sequence alignment of the GC3906 DNA sequence query to top DNA hits. Arrows represent the binding sites for the forward and reverse primers designed for this gene cluster in 5' to 3' and 3' to 5' orientation, respectively. Red bars represent nucleotide substitutions, white bars represent gaps, and blue 'I' symbols represent insertions. Whole genomes of *Xhv* strains submitted by Rosenthal et al., 2022 include those with sequence IDs starting with 'JAJTZ.'

A

Query	1	MPKPKTPATVYGTRLRHARMAMGWTQAELAERIGMVDSVSGATRVSRYETGQHDPDPATA	180	Matches:
X. hortorum pv. *vitians*	1	MPKPKTPATVYGTRLRHARMAMGWTQAELAERIGMVDSVSGATRVSRYETGQHDPDPATA	60	HTH transcriptional regulator (100%)
X. hortorum pv. *vitians*	21	MPKPKTPATVYGTRLRHARMAMGWTQAELAERIGMVDSVSGATRVSRYETGQHDPDPATA	80	HTH transcriptional regulator (100%)
X. hortorum	1	MPKPKTPATVYGTRLRHARMAMGWTQAELAERIGMVDSVSGATRVSRYETGQHDPDPATA	60	HTH transcriptional regulator (92.3%)
X. hortorum pv. *vitians*	21	MPKPKTPATVYGTRLRHARMAMGWTQAELAERIGMVDSVSGATRVSRYETGQHDPDPATA	80	Hypothetical protein (92.3%)
X. hortorum	21	MPKPKTPATVYGTRLRHARMAMGWTQAELAERIGMVDSVSGATRVSRYETGQHDPDPATA	80	HTH transcriptional regulator (85.5%)

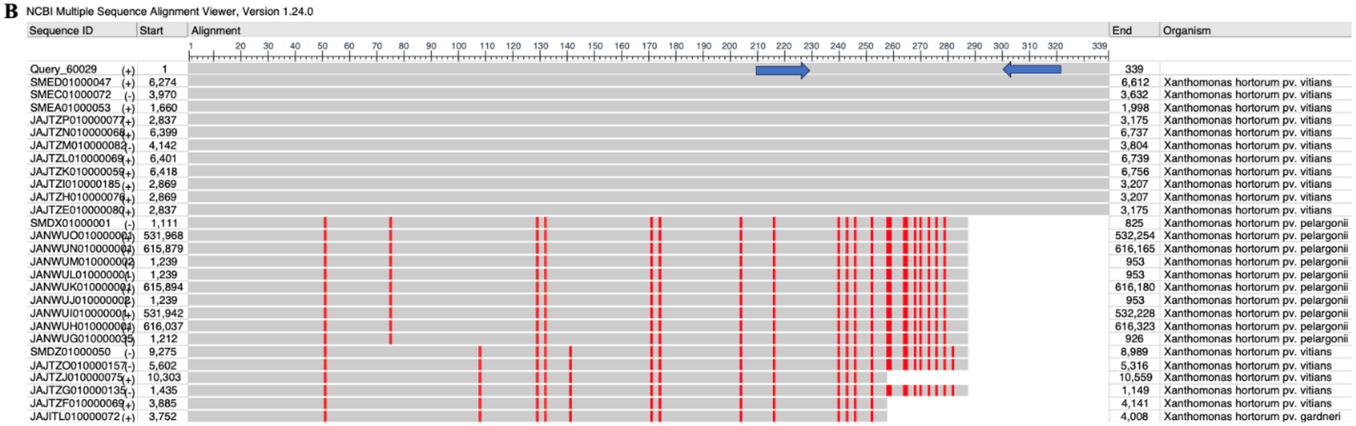

Figure S3. BLASTx protein alignments for top hits (A) and BLASTn DNA alignments (B) to query GC4021 sequence. (A) The top five protein matches to the GC4021 query DNA sequence; percent identity value for each match is shown in parentheses. **(B)** Multiple sequence alignment of the GC4021 DNA sequence query to top DNA hits. Arrows represent the binding sites for the forward and reverse primers designed for this gene cluster in 5' to 3' and 3' to 5' orientation, respectively. Red bars represent nucleotide substitutions. Whole genomes of *Xhv* strains submitted by Rosenthal et al., 2022 include those with sequence IDs starting with 'JAJTZ.'

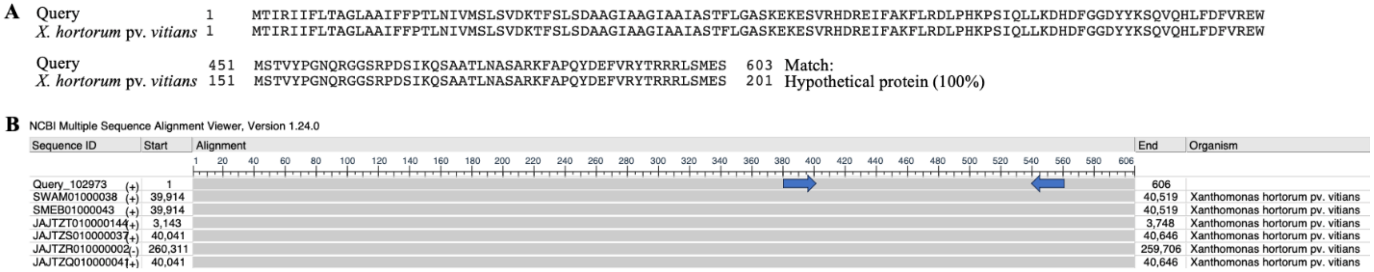

Figure S4. BLASTx protein alignments for top hits (A) and BLASTn DNA alignments (B) to query GC4381 sequence. (A) The singular protein match to the GC4381 query DNA sequence; percent identity value for the match is shown in parentheses. **(B)** Multiple sequence alignment of the GC4381 DNA sequence query to top DNA hits. Arrows represent the binding sites for the forward and reverse primers designed for this gene cluster in 5' to 3' and 3' to 5' orientation, respectively. Whole genomes of *Xhv* strains submitted by Rosenthal et al., 2022 include those with sequence IDs starting with 'JAJTZ.'

A

```
Query                  1     MPPGKSWLTQKPTLSIMPNHADYWHVFKEPHQATEYNQNSKARFALTGDSRGMFYVGDTLNGVLWETVLRDVEPNRRLEVDVDVNQLRDMRAVRLALMEPALPLLDLR
X. hortorum pv. vitians 1     MPPGKSWLTQKPTLSIMPNHADYWHVFKEPHQATEYNQNSKARFALTGDSRGMFYVGDTLNGVLWETVLRDVEPNRRLEVDVDVNQLRDMRAVRLALMEPALPLLDLR

Query                  451   SGFGMPGMPCLAWVSRQHSGSNVFLCYDPPTKPDNWLIKDNVVALDDPRTGHDLIKKALEEAGFSWAPAYYTSAI   675  Match:
X. hortorum pv. vitians 151   SGFGMPGMPCLAWVSRQHSGSNVFLCYDPPTKPDNWLIKDNVVALDDPRTGHDLIKKALEEAGFSWAPAYYTSAI   225  Hypothetical protein (100%)
```

B

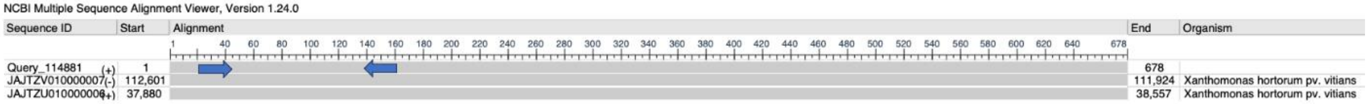

Figure S5. BLASTx protein alignments for top hits (A) and BLASTn DNA alignments (B) to query GC4980 sequence. (A) The top five protein matches to the GC4980 query DNA sequence; percent identity value for each match is shown in parentheses. (B) Multiple sequence alignment of the GC4980 DNA sequence query to top DNA hits. Arrows represent the binding sites for the forward and reverse primers in 5’ to 3’ and 3’ to 5’ orientation, respectively.

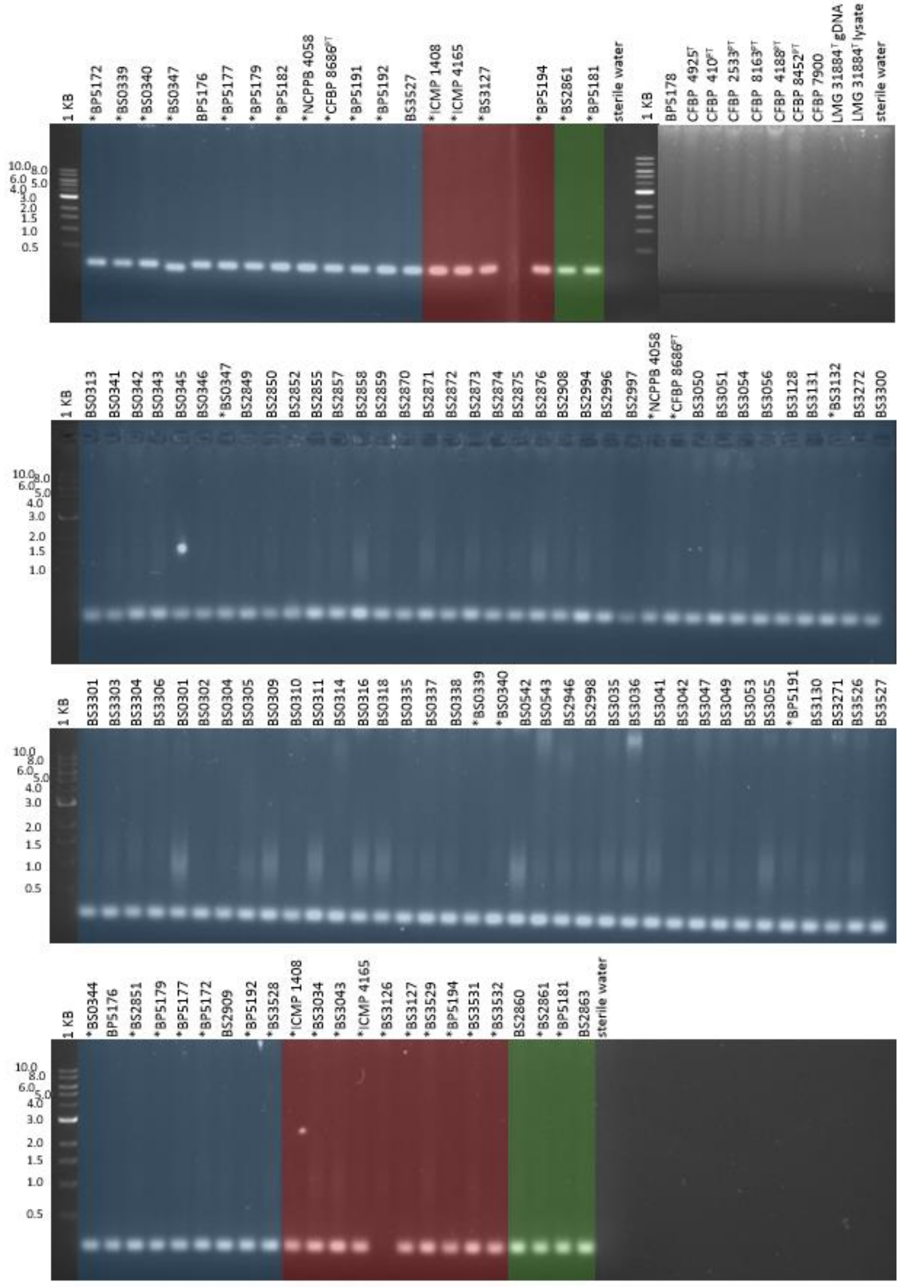
1 KB
*BP5172
*BS0339
*BS0340
*BS0347
BP5176
*BP5177
*BP5179
*BP5182
*NCPPB 4058
*CFBP 8686PT
*BP5191
*BP5192
BS3527
*ICMP 1408
*ICMP 4165
*BS3127
*BP5194
*BS2861
*BP5181
sterile water
1 KB
BP5178
CFBP 4925T
CFBP 410PT
CFBP 2533PT
CFBP 8163PT
CFBP 4188PT
CFBP 8452PT
CFBP 7900
LMG 31884T gDNA
LMG 31884T lysate
sterile water
10.0 8.0 6.0 5.0 4.0 3.0 2.0 1.5 1.0 0.5
1 KB
BS0313
BS0341
BS0342
BS0343
BS0345
BS0346
*BS0347
BS2849
BS2850
BS2852
BS2855
BS2857
BS2858
BS2859
BS2870
BS2871
BS2872
BS2873
BS2874
BS2875
BS2876
BS2908
BS2994
BS2996
BS2997
*NCPPB 4058
*CFBP 8686PT
BS3050
BS3051
BS3054
BS3056
BS3128
BS3131
*BS3132
BS3272
BS3300
10.0 8.0 6.0 5.0 4.0 3.0 2.0 1.5 1.0
1 KB
BS3301
BS3303
BS3304
BS3306
BS0301
BS0302
BS0304
BS0305
BS0309
BS0310
BS0311
BS0314
BS0316
BS0318
BS0335
BS0337
BS0338
*BS0339
*BS0340
BS0542
BS0543
BS2946
BS2998
BS3035
BS3036
BS3041
BS3042
BS3047
BS3049
BS3053
BS3055
*BP5191
BS3130
BS3271
BS3526
BS3527
10.0 8.0 6.0 5.0 4.0 3.0 2.0 1.5 1.0 0.5
1 KB
*BS0344
BP5176
*BS2851
*BP5179
*BP5177
*BP5172
BS2909
*BP5192
*BS3528
*ICMP 1408
*BS3034
*BS3043
*ICMP 4165
*BS3126
*BS3127
*BS3529
*BP5194
*BS3531
*BS3532
BS2860
*BS2861
*BP5181
BS2863
sterile water
10.0 8.0 6.0 5.0 4.0 3.0 2.0 1.5 1.0 0.5

Figure S6. *Xhv*-specific detection using the touchdown PCR method and the GC3906-152 primer set. Gel electrophoresis showing the bands produced following amplification of the target 152 bp fragment. The first row includes a preliminary test using a set of genomic DNA extracts and the bottom three rows represent a test of a larger *Xhv* collection using colony suspensions. Race 1 strains have a blue overlay, race 2 strains have a red overlay, and race 3 strains have a green overlay. The asterisks refer to *Xhv* strains of known races, determined by HR screening, while all other *Xhv* strains are hypothesized to belong to a race based on MLSA sequetype [10, 11] (Table 2). Non-target, closely related pathovars that were included to evaluate the specificity of this protocol were *X. hortorum* isolated from radicchio (BP5178), *X. hortorum* pv. *hederae* (CFBP 4925^{PT}), *X. hortorum* pv. *taraxaci* (CFBP 410^{PT}), *X. hortorum* pv. *pelargonii* (CFBP 2533^{PT}), *X. hortorum* pv. *gardneri* (CFBP 8163^{PT}), *X. hortorum* pv. *cynarae* (CFBP4188^{PT}), *X. hortorum* pv. *carotae* (CFBP 7900), and *X. campestris* pv. *coriandri* (CFBP 8452^{PT}). The size reference for the amplicons was a 1 KB ladder from New England Biolabs (NEB), and the 1% agarose gel was run for 2 hours at 84 V.

Formatted: Indent: Left: 0 cm

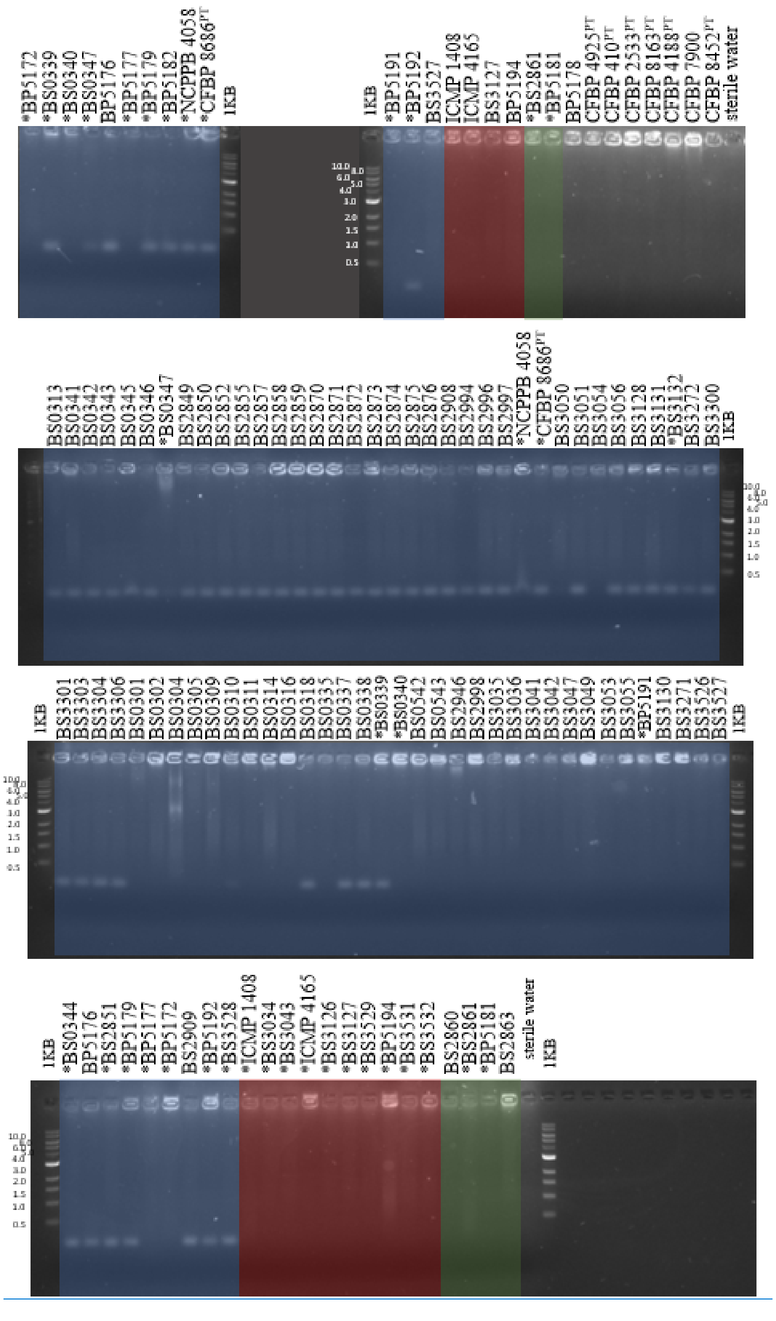
*BP5172
*BS0339
*BS0340
*BS0347
BP5176
*BP5177
*BP5179
*BP5182
*NCPPB 4058
*CFBP 8686PT
1KB
1KB
*BP5191
*BP5192
BS3527
ICMP 1408
ICMP 4165
BS3127
BP5194
*BS2861
*BP5181
BP5178
CFBP 4925PT
CFBP 410PT
CFBP 2533PT
CFBP 8163PT
CFBP 4188PT
CFBP 7900
CFBP 8452PT
sterile water
10.0
8.0
6.0
5.0
4.0
3.0
2.0
1.5
1.0
0.5
BS0313
BS0341
BS0342
BS0343
BS0345
BS0346
*BS0347
BS2849
BS2850
BS2852
BS2855
BS2857
BS2858
BS2859
BS2870
BS2871
BS2872
BS2873
BS2874
BS2875
BS2876
BS2908
BS2994
BS2996
BS2997
*NCPPB 4058
*CFBP 8686PT
BS3050
BS3051
BS3054
BS3056
BS3128
BS3131
*BS3132
BS3272
BS3300
1KB
1KB
BS3301
BS3303
BS3304
BS3306
BS0301
BS0302
BS0304
BS0305
BS0309
BS0310
BS0311
BS0314
BS0316
BS0318
BS0335
BS0337
BS0338
*BS0339
*BS0340
BS0542
BS0543
BS2946
BS2998
BS3035
BS3036
BS3041
BS3042
BS3047
BS3049
BS3053
BS3055
*BP5191
BS3130
BS3271
BS3526
BS3527
1KB
1KB
*BS0344
BP5176
*BS2851
*BP5179
*BP5177
*BP5172
BS2909
*BP5192
*BS3528
*ICMP 1408
*BS3034
*BS3043
*ICMP 4165
*BS3126
*BS3127
*BS3529
*BP5194
*BS3531
*BS3532
BS2860
*BS2861
*BP5181
BS2863
sterile water
1KB

Figure S7. Partial *Xhv* race 1 detection using the touchdown PCR method and the GC4021-112 primer set. Gel electrophoresis showing the bands produced following amplification of the target 112 bp fragment. The first row includes a preliminary test using a set of genomic DNA extracts and the bottom three rows represent a test of our larger *Xhv* collection using colony suspensions. Race 1 strains have a blue overlay, race 2 strains have a red overlay, and race 3 strains have a green overlay. The asterisks refer to *Xhv* strains of known races, determined by HR screening, while all other *Xhv* strains are hypothesized to belong to a race based on MLSA sequetype [10, 11] (Table 2). Non-target, closely related strains that were included to evaluate the specificity of our protocol were *X. hortorum* isolated from radicchio (BP5178), *X. hortorum* pv. *hederae* (CFBP 4925[PT]), *X. hortorum* pv. *taraxaci* (CFBP 410[PT]), *X. hortorum* pv. *pelargonii* (CFBP 2533[PT]), *X. hortorum* pv. *gardneri* (CFBP 8163[PT]), *X. hortorum* pv. *cynarae* (CFBP4188[PT]), *X. hortorum* pv. *carotae* (CFBP 7900), and *X. campestris* pv. *coriandri* (CFBP 8452[PT]). Our size reference was a 1 KB ladder from NEB, and the 1 % agarose gel was run for 2 hours at 84 V.

Commented [CB1]: add Sandoya and explain more clearly.

Formatted: Font: Not Bold

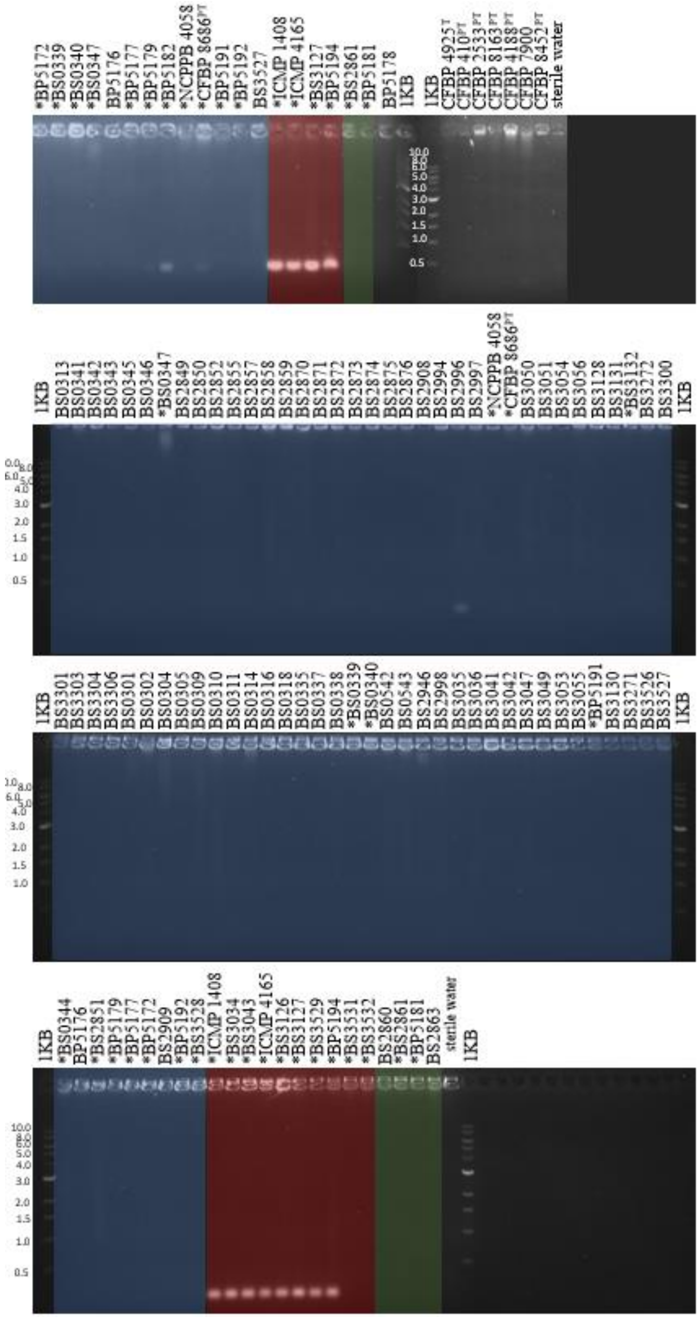
*BP5172
*BS0339
*BS0340
*BS0347
BP5176
*BP5177
*BP5179
*BP5182
*NCPPB 4058
*CFBP 8686PT
*BP5191
*BP5192
BS3527
*ICMP 1408
*ICMP 4165
*BS3127
*BP5194
*BS2861
*BP5181
BP5178
1KB
1KB
CFBP 4925T
CFBP 410PT
CFBP 2533PT
CFBP 8163PT
CFBP 4188PT
CFBP 7900
CFBP 8452PT
sterile water
10.0 8.0 6.0 5.0 4.0 3.0 2.0 1.5 1.0 0.5
1KB
BS0313
BS0341
BS0342
BS0343
BS0345
BS0346
*BS0347
BS2849
BS2850
BS2852
BS2855
BS2857
BS2858
BS2859
BS2870
BS2871
BS2872
BS2873
BS2874
BS2875
BS2876
BS2908
BS2994
BS2996
BS2997
*NCPPB 4058
*CFBP 8686PT
BS3050
BS3051
BS3054
BS3056
BS3128
BS3131
*BS3132
BS3272
BS3300
1KB
1KB
BS3301
BS3303
BS3304
BS3306
BS0301
BS0302
BS0304
BS0305
BS0309
BS0310
BS0311
BS0314
BS0316
BS0318
BS0335
BS0337
BS0338
*BS0339
*BS0340
BS0542
BS0543
BS2946
BS2998
BS3035
BS3036
BS3041
BS3042
BS3047
BS3049
BS3053
BS3055
*BP5191
BS3130
BS3271
BS3526
BS3527
1KB
1KB
*BS0344
BP5176
*BS2851
*BP5179
*BP5177
*BP5172
BS2909
*BP5192
*BS3528
*ICMP 1408
*BS3034
*BS3043
*ICMP 4165
*BS3126
*BS3127
*BS3529
*BP5194
*BS3531
*BS3532
BS2860
*BS2861
*BP5181
BS2863
sterile water
1KB
10.0 8.0 6.0 5.0 4.0 3.0 2.0 1.5 1.0 0.5

Figure S8. *Xhv* race 2 detection using the touchdown PCR method and the GC4381-178 primer set. Gel electrophoresis showing the bands produced following amplification of the target 178 bp fragment. The first row includes a preliminary test using a set of genomic DNA extracts and the bottom three rows represent a test of a larger collection of *Xhv* strains using colony suspensions. Race 1 strains have a blue overlay, race 2 strains have a red overlay, and race 3 strains have a green overlay. The asterisks refer to *Xhv* strains of known races, based determined by HR screening, while all other *Xhv* strains are hypothesized to belong to a race based on MLSA sequetype [10, 11] (Table 2). Non-target, closely related strains that were included to evaluate the specificity of our protocol were *X. hortorum* isolated from radicchio (BP5178), *X. hortorum* pv. *hederae* (CFBP 4925^{PT}), *X. hortorum* pv. *taraxaci* (CFBP 410^{PT}), *X. hortorum* pv. *pelargonii* (CFBP 2533^{PT}), *X. hortorum* pv. *gardneri* (CFBP 8163^{PT}), *X. hortorum* pv. *cynarae* (CFBP4188^{PT}), *X. hortorum* pv. *carotae* (CFBP 7900), and *X. campestris* pv. *coriandri* (CFBP 8452^{PT}). Our size reference for the amplicions was a 1 KB ladder from NEB, and the 1 % agarose gel was run for 2 hours at 84 V.

Formatted: Indent: Left: 0.32 cm, Right: 0.75 cm

Formatted: Font: (Asian) Chinese (Simplified, Mainland China)

Formatted: Indent: Left: 0 cm

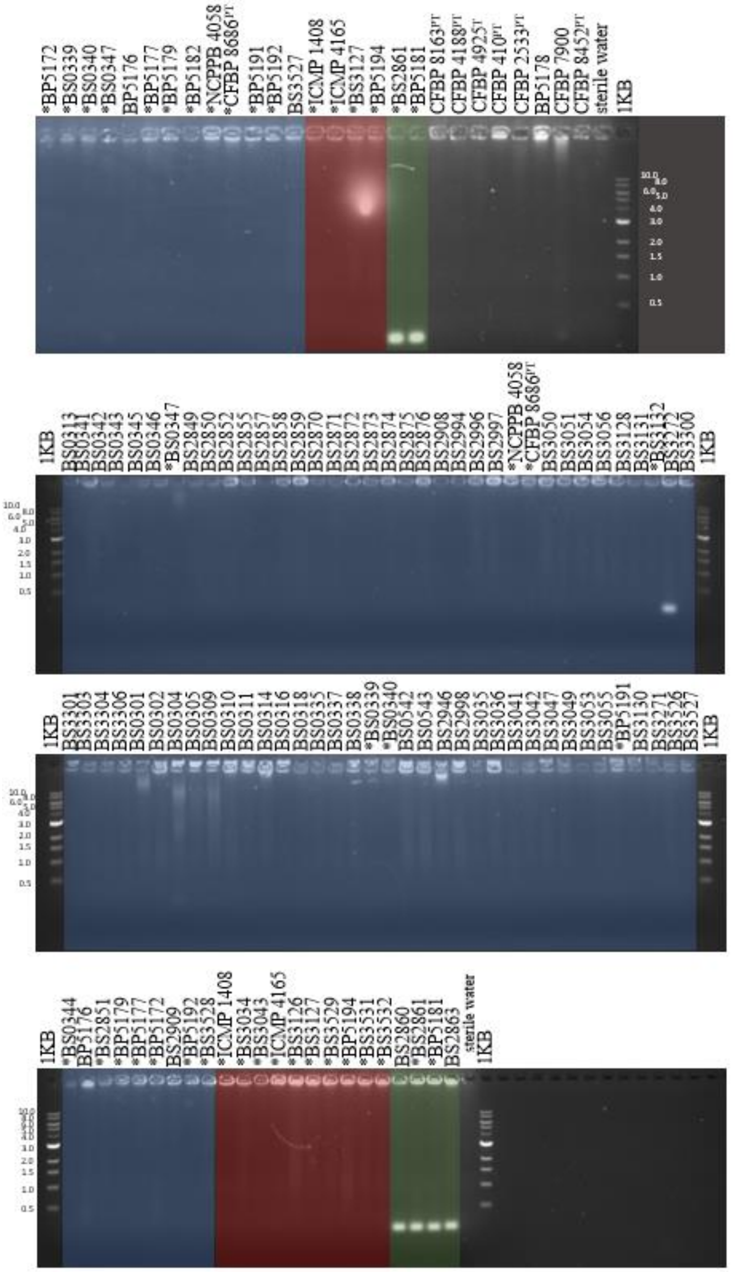

Figure S9. *Xhv* race 3 detection using the touchdown PCR method and the GC4980-138 primer set. Gel electrophoresis showing the bands produced following amplification of the target 138 bp fragment. The first row includes a preliminary test using a set of genomic DNA extracts and the bottom three rows represent a test of a larger *Xhv* collection using colony suspensions. Race 1 strains have a blue overlay, race 2 strains have a red overlay, and race 3 strains have a green overlay. The asterisks refer to *Xhv* strains of known races, based determined by HR screening, while all other *Xhv* strains are hypothesized to belong to a race based on MLSA sequetype [10, 11] (Table 2). Non-target, closely related strains that were included to evaluate the specificity of our protocol were *X. hortorum* isolated from radicchio (BP5178), *X. hortorum* pv. *hederae* (CFBP 4925[PT]), *X. hortorum* pv. *taraxaci* (CFBP 410[PT]), *X. hortorum* pv. *pelargonii* (CFBP 2533[PT]), *X. hortorum* pv. *gardneri* (CFBP 8163[PT]), *X. hortorum* pv. *cynarae* (CFBP4188[PT]), *X. hortorum* pv. *carotae* (CFBP 7900), and *X. campestris* pv. *coriandri* (CFBP 8452[PT]). Our size reference for the amplicions was a 1 KB ladder from NEB, and the 1 % agarose gel was run for 2 hours at 84 V.

Formatted: Indent: Left: 0.32 cm

Formatted: Indent: Left: 0.75 cm
